# Lipopolysaccharide promotes metastasis via acceleration of glycolysis by the nuclear factor-κB/snail/hexokinase3 signaling axis in colorectal cancer

**DOI:** 10.1186/s40170-021-00260-x

**Published:** 2021-05-12

**Authors:** Xuesong Wu, Senmi Qian, Jun Zhang, Jieqiong Feng, Ke Luo, Lichao Sun, Liang Zhao, Yuliang Ran, Liang Sun, Jing Wang, Fangying Xu

**Affiliations:** 1 Department of Pathology and Pathophysiology, and Department of General Surgery of the Second Affiliated Hospital, Zhejiang University School of Medicine, Hangzhou, People’s Republic of China; 2 Key Laboratory of Disease Proteomics of Zhejiang Province, Zhejiang University School of Medicine, Hangzhou, People’s Republic of China; 3 Present Address: Department of Pathology, the First Affiliated Hospital, Zhejiang University Medical School, Hangzhou, People’s Republic of China; 4 Department of Pathology, Zhejiang Provincial Hospital of Chinese Medicine, Hangzhou, People’s Republic of China; 5 Department of Pathology and Pathophysiology, Zhejiang University School of Medicine, Hangzhou, People’s Republic of China; 6 State Key Laboratory of Molecular Oncology, National Cancer Center/Cancer Hospital, Chinese Academy of Medical Sciences and Peking Union Medical College, Beijing, People’s Republic of China; 7 Department of Pharmacy, Shanghai Baoshan Luodian Hospital, Shanghai, People’s Republic of China

**Keywords:** Lipopolysaccharide, Inflammasome, Caspase-1, NF-κB, Snail, Hexokinase 3, Colorectal cancer, Metastasis, Glycolysis, Metformin

## Abstract

**Background:**

Cancer cell is generally characterized by enhanced glycolysis. Inflammasome activation is interaction with glycolysis. The concentration of lipopolysaccharide (LPS), a classic inflammasome activator, is significantly higher in colorectal cancer tissue than in normal intestinal mucosa. However, the mechanism of LPS on glycolysis and metastasis has not been fully elucidated. This study aimed to investigate the roles of LPS on inflammasome activation, glycolysis, and metastasis, and unravel metformin’s potential in treatment of CRC.

**Methods:**

We detected inflammasome activation and cell motility following LPS exposure in CRC cell lines. Glycolysis analysis was performed, and the key glycolytic rate-limiting enzymes were detected. Dual-luciferase reporter gene assay, co-immunoprecipitation, chromatin immunoprecipitation (ChIP) analysis, and ChIP-reChIP assay were performed to identify the specific mechanisms of LPS on glycolysis. Mouse metastasis models were used to determine the effects of LPS and metformin on metastasis. Correlation analysis of the expression of various molecules was performed in 635 CRC samples from The Cancer Genome Atlas and 83 CRC samples from our lab.

**Results:**

LPS activates caspase-1 through NF-κB and upregulates the expression of Snail and HK3 depending on caspase-1 activation. LPS potentiates migration and invasion depending on accelerated glycolysis, which could be reversed by knockdown of glycolytic rate-limiting enzyme HK3. Nuclear Snail is upregulated by NF-κB under LPS treatment and then forms a complex with NF-κB, then directly binds to the HK3 promoter region to upregulate the expression of HK3. Metformin suppresses the NF-κB/Snail/HK3 signaling axis that is activated by LPS and then inhibits LPS-induced metastasis. In vivo, LPS-treated cells form more metastasis in the lungs of mice, and metformin completely reverses this effect of LPS.

**Conclusion:**

LPS activates inflammasomes in cancer cells through NF-κB and promotes metastasis through glycolysis enhanced by the NF-κB/Snail/HK3 signaling pathway in CRC. Metformin could prevent this effect of LPS.

**Supplementary Information:**

The online version contains supplementary material available at 10.1186/s40170-021-00260-x.

## Background

Microorganisms drive the initiation and progression of approximately 15–20% human cancers [1]. There are approximately 100 trillion bacteria in the intestine, including >1000 different species [2]. Recently, researchers have focused on the relationship between the gut microbiota and cancer, including modulation of the release of inflammatory mediators, alterations of the immune microenvironment, and interaction with metabolism [3–5].

LPS, characteristic components of the cell wall of Gram-negative bacteria, is a classic inflammasome activator. In intestinal epithelial cells, internal LPS induces inflammasome activation through initiating the Toll-like receptor 4 (TLR4)-mediated signal transduction cascade [6]. Inflammasome activation is characterized by proteolytic processing of pro-interleukin-1β (pro-IL-1β) and pro-IL-18 by caspase-1 into mature IL-1β and IL-18. Patients with CRC have higher concentration of LPS than healthy persons both in blood and CRC tissues [7, 8]. However, most studies on LPS were performed using immune cells, whereas its effects on cancer cells remain to be elucidated.

LPS induces macrophage inflammation and injuries through transcription factor nuclear factor-κB (NF-κB). P65, a NF-κB subunit, accumulates in the nucleus and can recruit nuclear Snail by cooperatively binding to the promoters of a cluster of genes [9]. P65 stabilizes Snail and promotes invasion and migration of breast cancer cells after tumor necrosis factor (TNF) α treatment [10]. Snail regulates glucose flux by repressing phosphofructokinase, platelet (PFKP) and fructose-1,6-biphosphatase (FBP1) [11, 12]. NF-κB also affects glucose metabolism, for example, it regulates the transcription of hexokinase 2 (HK2) in macrophages [13].

Recent studies have demonstrated that glycolytic enzymes and inflammasomes can interact with each other. Hexokinase 1 (HK1), the enzyme catalyzing the first step in the glycolytic pathway, triggers NLRP3 inflammasome activation [14]. NLRP3 inflammasome activation and subsequent IL-1β release modulate glycolysis through 6-phosphofructo-2-kinase/fructose-2,6-bisphosphatase 3 (PFKFB3) [15]. The Warburg effect (cancer cells tending to favor metabolism via glycolysis even in aerobic conditions) has been verified and characterized in tumors. Increased glucose consumption and lactate production are features of glycolysis upregulation. However, how LPS regulates cancer cell motility through glycolysis needs further research.

Hexokinases (HKs) are glycolytic rate-limiting enzymes, with four isozymes in humans, including HK1, HK2, HK3, and HK4 (glucokinase). HK3 has lower protein expression but higher glucose affinity than the others. HK3 expression is upregulated in CRC tissue compared with that in normal tissue and is positively correlated with some metastasis-related genes [16]. The role and mechanism of HK3 needs to be investigated further.

Metformin directly inhibits the enzymatic activity of hexokinase and reduces cell glucose consumption and tumor growth in breast cancer [17]. LPS injures the intestinal barrier and induces inflammation; however, metformin treatment can ameliorate the damage by inhibiting NF-κB phosphorylation [18]. Metformin can also inhibit inflammasome activation through AMP-activated protein kinase (AMPK) [19]. In human intestinal epithelium, metformin reaches a higher concentration than in other tissues and changes the composition of gut microbiota [20, 21].

In this study, we aimed to reveal the action of LPS on inflammasome activation and glycolysis in cancer cells, the mechanism underlying metastasis, and the role of metformin in preventing metastasis in CRC.

## Materials and methods

RNA interference, real-time PCR, Western blotting, plasmid construction and dual-luciferase reporter assay, chromatin immunoprecipitation (ChIP) analysis, cell migration and invasion assay, CCK8 assay, and flow cytometry analysis are described in the Supplementary file 1.

### Cell culture and reagents

The human CRC cell lines DLD1 and RKO were purchased from the ATCC in 2016 and cultured in RPMI 1640 supplemented with 10% fetal bovine serum. HEK293T was purchased from the cell bank at the Chinese Academy of Sciences (Shanghai, China) in 2016 and cultured in DMEM supplemented with 10% FBS. All cell lines have no mycoplasma contamination and were identified by short tandem repeat (STR)-based methods. Cells were frozen at low passage and used within 25 passages after thawing. Cells were treated with LPS for inflammasome activation and Ac-YVAD-CHO for caspase-1 inhibition. Bay11-7082 or JSH-23 was used for NF-κB inhibition.

### Caspase-1 enzyme activity measurement

Caspase-Glo®1 inflammasome assay kit (Promega, WI, USA) was used. Briefly, after removing cell culture medium, an aliquot of 100μl of the Caspase-Glo 1 reagent was added and mixed. The mixture was then incubated for 1 h at room temperature before measuring the luminescence on a Thermo Technologies Luminometer.

### 2-NBDG uptake assay

Cells were plated, and cell culture mediums were replaced by glucose-free medium in the next day. Two hours before the end of LPS treatment, 0.3mM of 2-NBDG ((2-(N-(7-Nitrobenz-2-oxa-1,3-diazol-4-yl)Amino)-2-Deoxyglucose)) (Invitrogen, CA, USA) was added into each well at 37 °C. Images were captured using Zeiss Axio Observer A1 microscope, and integrate optical density was calculated by Image Pro Plus 6.0 (Media Cybernetics, Washington D.C., USA)

### Glucose consumption and lactate production assays

The glucose and lactate concentrations in cell medium supernatants were measured with a commercial glucose test kit GAGO20 (Sigma-Aldrich, St Louis, USA) and lactic acid assay kit (Megazyme, Ireland) respectively. Briefly, cells grew in DMEM containing 5 mM glucose. Culture medium was collected at 1, 2, 4, 8, 16, and 24 h after incubation. For lactate level detection, all samples were diluted to yield a lactic acid concentration of 0.03–0.30 g/L, and added with reagent in the kit to form a 224 μl reaction volume to detect the absorbance.

### Co-immunoprecipitation (co-IP)

Cells were lysed in IP Lysis Buffer containing a protease inhibitor PMSF. Whole cell extracts were centrifuged; then, supernatants were collected and incubated with SureBeads™ Protein G Magnetic Beads (Bio-Rad Laboratories, CA, USA) together with specific antibodies. After overnight incubation at 4°C, protein G magnetic beads were washed with IP wash buffer PBS-T (PBS (phosphate buffer solution) + 0.1% Tween 20). Immunoprecipitates were eluted and boiled in 1× loading buffer and tested by western blotting. Antibodies used for Co-IP were anti-P65 (CST, MA, USA) and anti-Snail (CST, MA, USA).

### Immunofluorescence

Cells were fixed in 4% paraformaldehyde for 10 min and permeabilized with 0.5% Triton X-100 for 20 min at room temperature. Next, cells were blocked with 10% BSA in PBS for 1 h and incubated with primary antibodies against P65 (CST, Cat#4764, 1:200) and Snail (R&D, Cat# AF3639, 1:200) at 4°C overnight, then incubated with Alexa Fluor 594 AffiniPure Goat anti-rabbit (Yeasen, Cat#33112ES60, 1:200) and Alexa Fluor 488 AffiniPure Donkey anti-Goat (Yeasen, Cat#34306ES60, 1:200) secondary antibodies for 1 h at room temperature. Subsequently, the nuclei were stained with 4’,6-diamidino-2-phenylindole (DAPI) (1:5000, Beyotime). The intracellular distributions of P65 and Snail were finally observed by a laser scanning confocal microscopy (LSM 880) at a magnification of ×60.

### Chromatin immunoprecipitation (ChIP)-reChIP assays

ChIP-reChIP assays were performed by Re-ChIP-IT kit (Active Motif, Cat# 53016) following the manufacturer’s recommendations. Briefly, after first ChIP with P65 antibody (CST, Cat#8242), the protein-chromatin complex was divided equally into several aliquots. Samples not used for second ChIP were decrosslinked and purified by Proteinase K and phenol/chloroform extractions. The other aliquot of chromatin was subsequently used in the second ChIP reaction. Chromatin was washed, then eluted with a specific Re-ChIP-IT elution buffer. Next, chromatin from the first ChIP reaction was desalted and subjected to second ChIP with Snail antibody (R&D, Cat#AF3639). In addition, a control sequential ChIP was carried out in which chromatin was immunoprecipitated first with an antibody against P65 followed by no antibody. Aliquots of input DNA before and after performing the first ChIP reaction were used as controls in PCR analysis together with the second ChIP samples. As a negative control, rabbit IgG was used for non-specific ChIP. Re-ChIP chromatin level was determined by semi-quantitative PCR analysis. The PCR products were separated on a 1% agarose gel containing ethidium bromide for visualization and analysis.

### Clinical specimens

There were 83 tumor tissues for real-time PCR and 9 samples for western blotting. Total RNA was extracted from tissue samples using TRIzol reagent (Pufei, Shanghai, China) according to the manufacturer’s instructions. For mRNA quantification and real-time PCR, RNA was reverse transcribed to cDNA using PrimeScript™ RT reagent kit with gDNA Eraser (Takara, Tokyo, Japan). For protein extraction, whole protein extraction kit (KeyGen Biotech, Nanjing, China) was used following the manufacturer’s protocol. This study was approved by the Ethics Committee of Zhejiang University’s School of Medicine and was carried out in accordance with the Declaration of Helsinki.

### Bioinformatic data mining

We downloaded RNA-seq gene expression data and clinical data using UCSC Xena Browser (https://xenabrowser.net). The gene expression data of primary colon cancer and rectal cancer was based on the Cancer Genome Atlas–GDC TCGA colon cancer (TCGA-COAD, version 09-14-2017) and GDC TCGA rectal cancer (TCGA-READ, version 09-15-2017), then normalized with HTseq-FPKM-UQ calculation. There are total 635 samples, including 469 colon cancers and 166 rectal cancers.

### Mouse tail-vein assay

The experimental protocol was approved by the Animal Care and Use Committee of Zhejiang University. For pulmonary metastasis assay, RKO cells were first transfected with pEZ-Lv201 (GeneCopoeia, MD, USA) luciferase vector and then treated with puromycin at 2μg/ml to obtain a RKO cell line that stably expresses luciferase. Next, Nod Scid mice (male, 5 weeks) were divided into three groups. The stable cell line was then treated with 1μg/ml LPS for 24 h or with 5mM metformin for 25 h and 1μg/ml LPS for 24 h, or with nothing; then, cells were trypsinized and resuspended in a PBS solution at a density of 8×10^4^ cells/μl, and 100μl of the suspension was injected into the lateral tail vein of each mouse. All experiments were performed using five mice per treatment group.

Mice were examined for metastatic foci by bioluminescence imaging. Mice were first injected with luciferin (GoldBio, Cat#LUCK-1G, 300 mg/kg, 5 min prior to imaging), anesthetized with 3% isoflurane, and then imaged in an IVIS spectrum imaging system (Caliper, Newton, USA). Images were analyzed with the Living Image software (Caliper, MA, USA). Bioluminescent flux (photons/s/sr/cm^2^) was determined for the tumors.

In tail vein assay of cancer metastasis, the mice were sacrificed after 4 weeks, and the lungs were collected and fixed by formalin. After paraffin embedding and tissue slicing, slides were stained by hematoxylin and eosin (H&E). The number of metastatic lung nodules was counted under the light microscope.

In survival analysis, one mouse from the group of LPS and metformin treatment was still alive after 3 months and was euthanized at that point.

### Statistical analysis

Scatter plots were drawn, and Spearman correlation was conducted for gene expression data in tissue samples. Statistical significance between groups was determined by two-tailed Student’s *t*-test or one-way analysis of variance with S-N-K test for post hoc multiple comparisons. Survival plots were drawn by Kaplan-Meier methods, and comparison between survival plots was performed by log-rank test. Statistical analyses were performed using the IBM SPSS Statistics 24.0 software (IBM Corp, NY, USA) or GraphPad Prism; the *P*-value of less than 0.05 was considered to be statistically significant.

## Results

### LPS-induced inflammasome activation promotes metastasis in CRC

**Fig. 1:**
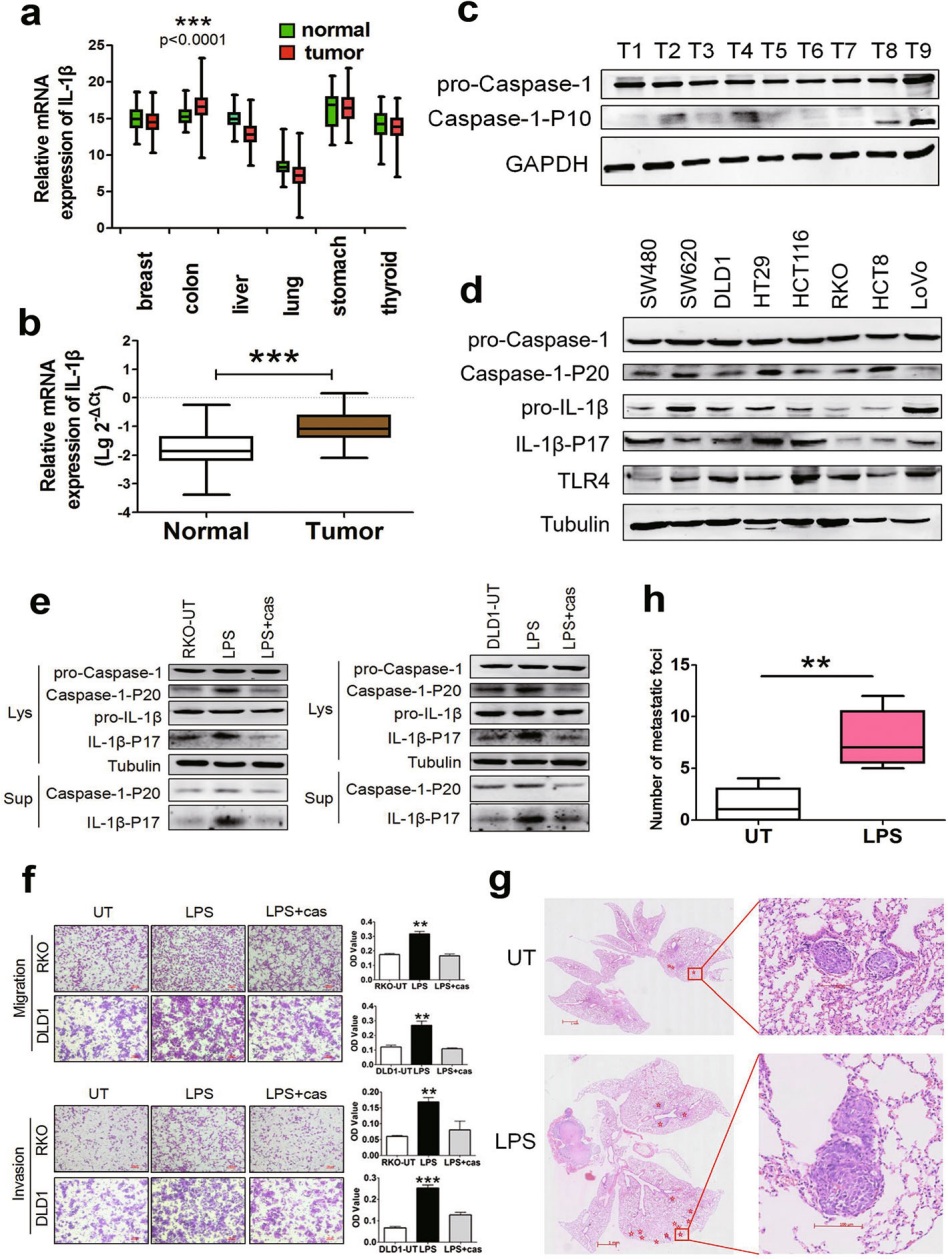
LPS-induced inflammasome activation promotes CRC progression. **a** Relative mRNA level of IL-1β in tumor and corresponding normal tissues of the breast, colon, liver, lung, stomach, and thyroid from TCGA dataset. **b** Relative mRNA level of IL-1β in tumor and matched normal tissues from 83 CRC cases. mRNA level was determined by real-time PCR, and logarithmic transformed data were used. **c** Inflammasome activation level in nine CRC tissue samples. Protein expression was determined by western blotting. **d** Detection of LPS receptor TLR4, and markers of inflammasome activation by western blotting in eight CRC cell lines, including SW480, SW620, DLD1, HT-29, HCT116, RKO, HCT8 and LoVo. **e** Western blotting was performed to detect Caspase-1-P20 and IL-1β-P17 expression in whole cell lysates (lys) and supernatants (Sup). Cells were pretreated with 3μM caspase-1 inhibitor Ac-YVAD-CHO (cas) for 3h, and then stimulated with 1μg/ml LPS for 24h. **f** LPS enhanced migration and invasion of CRC cells by transwell assay, which could be blocked by Ac-YVAD-CHO (cas). OD values were measured by a microplate reader at 570 nm. **g** Representative images (H&E staining) presented number of metastatic foci of lung in mice with tail-vein injection of untreated (UT) or LPS-treated RKO cells; the stars indicated the metastatic foci. **h** Statistical analysis of numbers of lung metastatic foci in mice. Data are presented as mean±SEM. *P < 0.05, **P < 0.01, ***P < 0.001

The mRNA expression of IL-1β in tumor and normal tissues from the cancer genome atlas (TCGA) dataset and 83 CRCs with matched normal samples in our laboratory were analyzed. In the TCGA data, among breast cancer, colon cancer, liver cancer, lung cancer, stomach cancer, and thyroid cancer, IL-1β expression was elevated in cancer tissues compared with that in matched normal tissues only in the colon (Fig. 1a). In our laboratory data, the expression of IL-1β was higher in CRC tissues than in normal tissues (Fig. 1b). Western blotting showed that the expression of pro-caspase-1 was similar, whereas the expression of caspase-1-P10 (activated caspase-1) varied among nine CRC tissue samples (Fig. 1c). These results suggest that inflammasome activation is associated with tumorigenesis or progression of CRC. Next, we detected the protein expression of LPS receptor TLR4, and inflammasome activation markers Caspase-1-P20 (activated caspase-1) and IL-1β-P17 (matured IL-1β) in eight CRC cell lines. Among these cell lines, RKO cells with lower level of Caspase-1-P20 and IL-1β-P17 and DLD1 cells with higher level of these markers were selected as in vitro models (Fig. 1d). To determine the optimal treatment condition, RKO cells were treated with LPS at 0, 1, 2, 4, and 8 μg/ml for 24 h. Western blotting and Caspase-Glo^®^ 1 Inflammasome Assay Kit were used to detect the protein expression of inflammasome activation markers and caspase-1 enzyme activity, respectively. At 1 μg/ml, LPS significantly activated inflammasomes (Supplementary Fig. 1a, b). Next, the cells were treated with 1 μg/mL LPS for various periods of time (Supplementary Fig. 1c). Thereafter, cells were treated with LPS for 24 h at 1 μg/ml in the subsequent cell experiments unless otherwise stated. Further, we found that LPS treatment promoted inflammasome activation and potentiated cell migration and invasive capacity, which could be reversed by the specific caspase-1 inhibitor Ac-YVAD-CHO (Fig. 1e, f). CCK8 assays and flow cytometry showed that there was no significant change in cell proliferation and apoptosis with or without LPS treatment (Supplementary Fig. 1d, e) and with or without caspase-1 inhibitor (Supplementary Fig. 2a, b). In vivo, RKO cells were injected into the tail vein of mice, and metastatic loci in the lungs were evaluated using H&E-stained slides. There were 1, 0, 0, 2, and 4 lung metastases in the untreated group, and 6, 7, 5, 9, and 12 lung metastases in the LPS group, respectively. The metastatic foci in the lungs of mice injected with LPS-treated cells were significantly more numerous than in mice without LPS treatment (Fig. 1g, h).

Cumulatively, LPS promotes cell motility and metastasis of CRC cells.

### LPS promotes inflammasome activation and cell movements through NF-κB

**Fig. 2:**
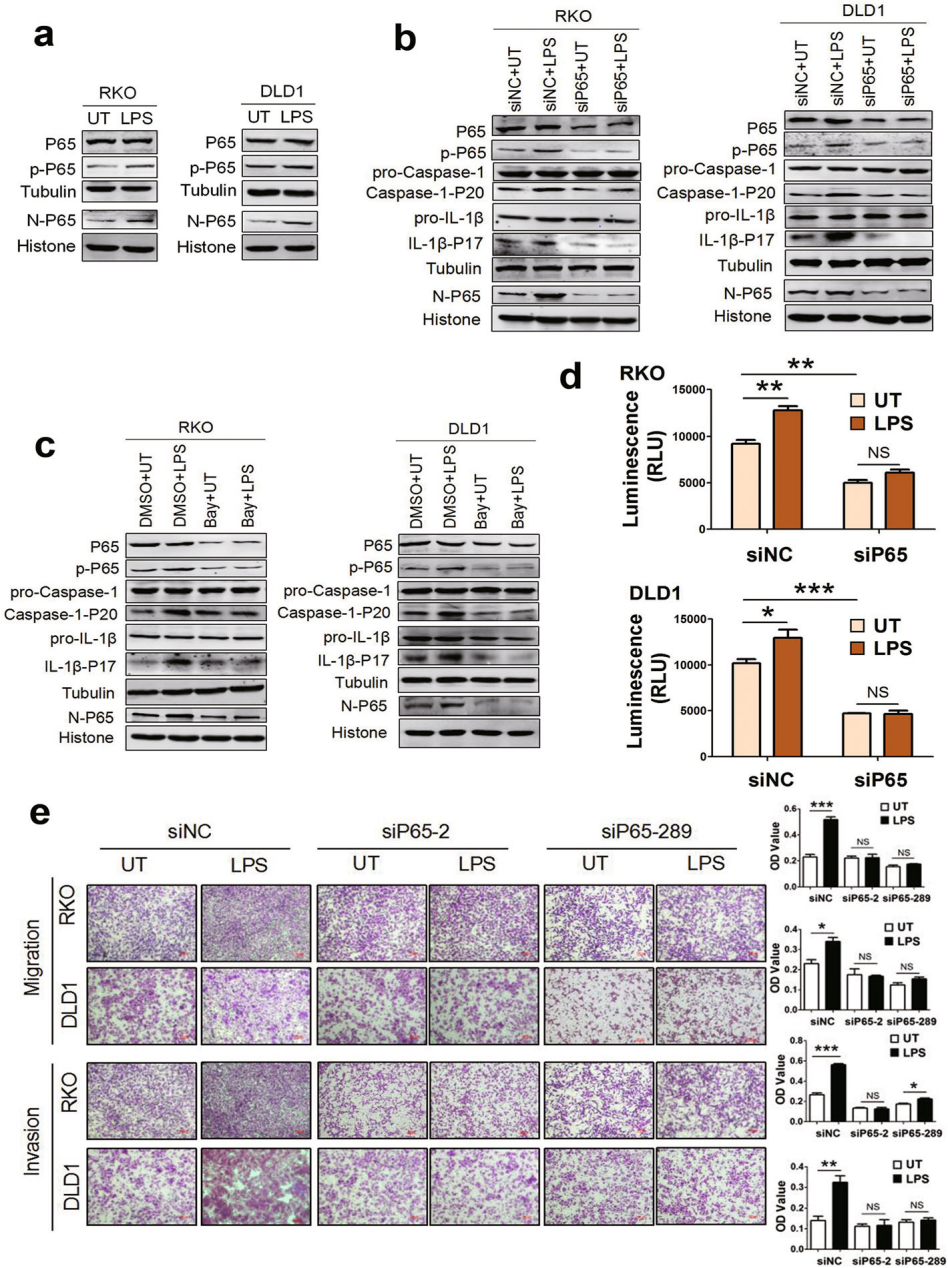
P65 is required for LPS-induced inflammasome activation and cell movements. **a** LPS induced P65 phosphorylation and transportation to nucleus. **b** Cells were transfected with siNC (control) or siP65 (knockdown of P65 with specific siRNA, 50nM for 48 h), then treated with 1μg/ml LPS for 24h. Phosphorylated P65, nuclear P65, and inflammasome activation makers Caspase-1-P20 and IL-1β-P17 were determined by western blotting. **c** To inhibit NF-κB activity, 50μM Bay11-7082 was added 3h prior to the addition of LPS. DMSO was solvent of Bay11-7082. Phosphorylated and nuclear P65 and inflammasome activation makers were determined by western blotting. **d** Cells were transfected with siNC (control) or siP65 for 48 h, then treated with LPS 1μg/ml for 24 h. Enzyme activity of caspase-1 was detected by Caspase-Glo^®^ 1 Inflammasome Assay Kit. **e** Transwell assay was performed to investigate migration and invasive properties of CRC cells with or without LPS treatment in the presence or absence of siP65 transfection. Data are presented as mean±SEM. *P<0.05, **P<0.01, ***P<0.001. N, nuclear; p-P65, phosphorylated P65; RLU: relative luminometer units

The transcription factor NF-κB has been implicated in tumor progression [22] and plays a central role in tumor metastasis by affecting inflammasome activation in immune cells [23]. We measured the effects of P65, the most widely studied member of NF-κB, on LPS-induced CRC inflammasome activation and CRC cell movements. We found that the levels of phosphorylated P65 and nuclear P65 significantly increased after LPS treatment (Fig. 2a). However, LPS-upregulated expression of phosphorylated P65, nuclear P65, and inflammasome activation markers was suppressed after knockdown of p65 with siRNA as well as inhibition of P65 with the specific NF-κB inhibitor Bay11-7082 (Fig. 2b, c). Caspase-1 enzyme activity assay showed that LPS promoted inflammasome activation in the presence of P65 (Fig. 2d). These results suggest that LPS induces inflammsome activation in a P65-dependent manner in CRC cells.

Moreover, transwell assay showed that LPS-induced migration and invasion were significantly inhibited by knockdown of P65 with siRNA, indicating that LPS enhanced CRC cell movements through P65 (Fig. 2e). CCK8 assays showed that there was no significant change in cell proliferation with or without P65 knockdown (Supplementary Fig. 2c, d).

### LPS upregulates snail expression through P65 and inflammasome activation to promote CRC cell movements

**Fig. 3:**
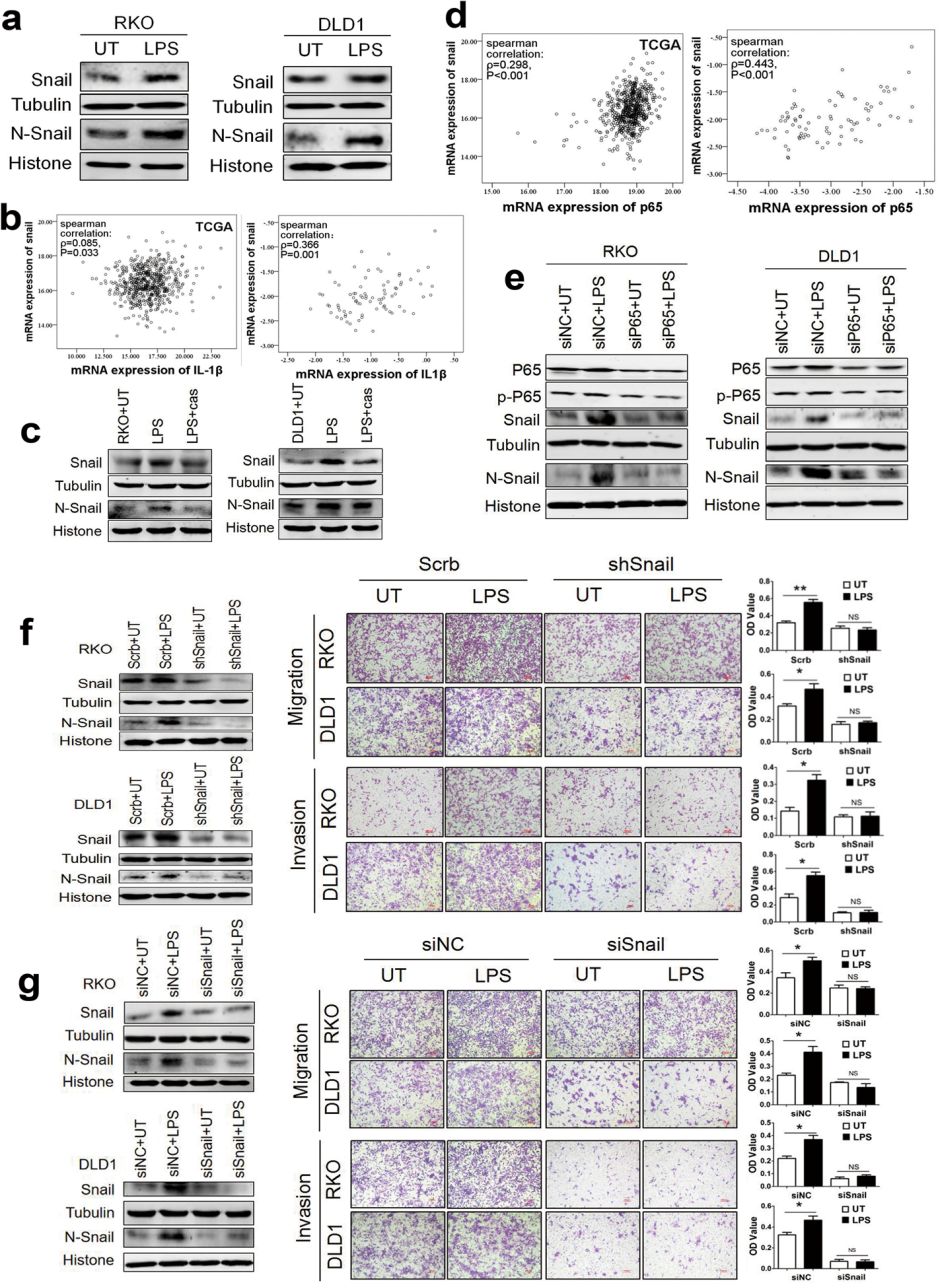
Snail is required for LPS-induced invasion and migration through P65/inflammasome activation. **a** The effects of LPS on Snail expression were examined by western blotting. **b** Scatter plot and spearman correlation analysis of Snail and IL-1β mRNA expression in TCGA database (left) and our data (right) of CRC respectively. Logarithmic transformed data were used in our tissue samples. *ρ* was Spearman’s rank correlation coefficient. **c** LPS promoted Snail expression and transportation to the nucleus through inducing inflammasome activation. Cells were pretreated with 3μM caspase-1 inhibitor Ac-YVAD-CHO (cas) for 3 h, and then stimulated with LPS 1μg/ml for 24 h. **d** Scatter plot and spearman correlation analysis of p65 and snail mRNA expression in TCGA database (left) and our tissue samples (right) of CRC. Logarithmic transformed data were used in our tissue samples. *ρ* was Spearman’s rank correlation coefficient. **e** The effects of P65 knockdown on Snail expression were examined by western blotting. Transient siRNA transfections were performed by transfecting 2 × 10^5^ RKO or DLD1 cells with 50nM control siRNA (siNC) or siRNA-P65 for 48 h, then treated with 1μg/ml LPS for 24 h. **f** Transwell assay was performed to investigate migration and invasion of CRC cells, and quantification of OD value was measured with microplate reader at 570 nm. Established Snail knockdown (shSnail) RKO and DLD1 cell lines were untreated or treated with 1μg/ml LPS. **g** Transwell assay was performed to investigate migration and invasion of CRC cells, and quantification of OD value was measured with microplate reader at 570 nm. Transient siRNA transfections were performed by transfecting 2 × 10^6^ RKO or DLD1 cells with 50nM control siNC or siRNA-Snail for 48 h, then untreated or treated with 1μg/ml LPS. Data are presented as mean±SEM. *P<0.05, **P<0.01, ***P<0.001. N, nuclear

Recently, a positive correlation between NF-κB and Snail activation has been described in several cancers [10]. To investigate whether LPS affects the expression of Snail, cells were treated with LPS, and Snail expression was detected using western blotting. The results showed that LPS could markedly upregulate Snail protein expression and Snail transportation to the nucleus (Fig. 3a). In addition, correlation analysis showed that IL-1β had a positive correlation with Snail in both 83 colorectal tissues and TCGA samples (Fig. 3b). To study the effect of LPS-induced inflammasome activation on Snail expression, we treated CRC cells with the caspase-1 inhibitor Ac-YVAD-CHO before LPS treatment. The results showed that pretreatment with Ac-YVAD-CHO could inhibit LPS effect on Snail upregulation, suggesting that LPS-induced inflammasome activation led to the upregulation of Snail expression (Fig. 3c). Moreover, the correlation analysis results showed that P65 positively correlated with Snail (Fig. 3d). To determine whether NF-κB activation was involved in LPS-mediated Snail expression, we transfected siRNA-P65 to RKO and DLD1 cells followed by LPS treatment and then detected Snail protein expression. Intriguingly, LPS no longer upregulated Snail expression after knockdown of P65, indicating that LPS promoted Snail expression by activating NF-κB signaling (Fig. 3e). These data strongly suggest that LPS upregulates Snail expression through NF-κB and inflammasome activation.

To examine the effect of Snail on the motility of CRC cells, transwell assay was performed using stable knockdown (shSnail) of DLD1 and RKO cell lines. We found that LPS promoted the migration and invasion of CRC cells in the control group (Scrb), whereas knockdown of Snail (shSnail) significantly prevented migration and invasion of LPS-treated CRC cells (Fig. 3f). Meanwhile, there was no significant change in cell proliferation when Snail was knockdown (Supplementary Fig. 2e, f). The results showed that Snail served as a critical factor in LPS-induced cell motility. Moreover, cells were transiently transfected with siRNA-Snail, and non-targeting siRNA was used as a control (siNC). After 48 h, the cells were trypsinized and plated in the upper chamber and treated with LPS for the next 24 h. The results showed that LPS could promote CRC cell migration and invasion in control group (siNC). However, LPS treatment no longer affected cell motility after knockdown of Snail, which indicated that Snail abundance was essential to LPS-induced CRC motility (Fig. 3g).

### LPS promotes glycolysis and cell motility and depends on HK3

Emerging studies indicate that the upregulation of glycolysis is an important feature of the malignant phenotype of invasive cancer [24]. To test whether glucose is required for the invasion and migration of CRC cells, we compared cell motility under glucose-starved or glucose-enriched condition. We found that LPS could promote migration and invasion of CRC cells only in a glucose-nourished environment but did not exert its activity in a glucose-starved condition (Supplementary Fig. 3). The results showed that LPS enhanced the migration and invasion of CRC cells depending on glucose uptake.

**Fig. 4:**
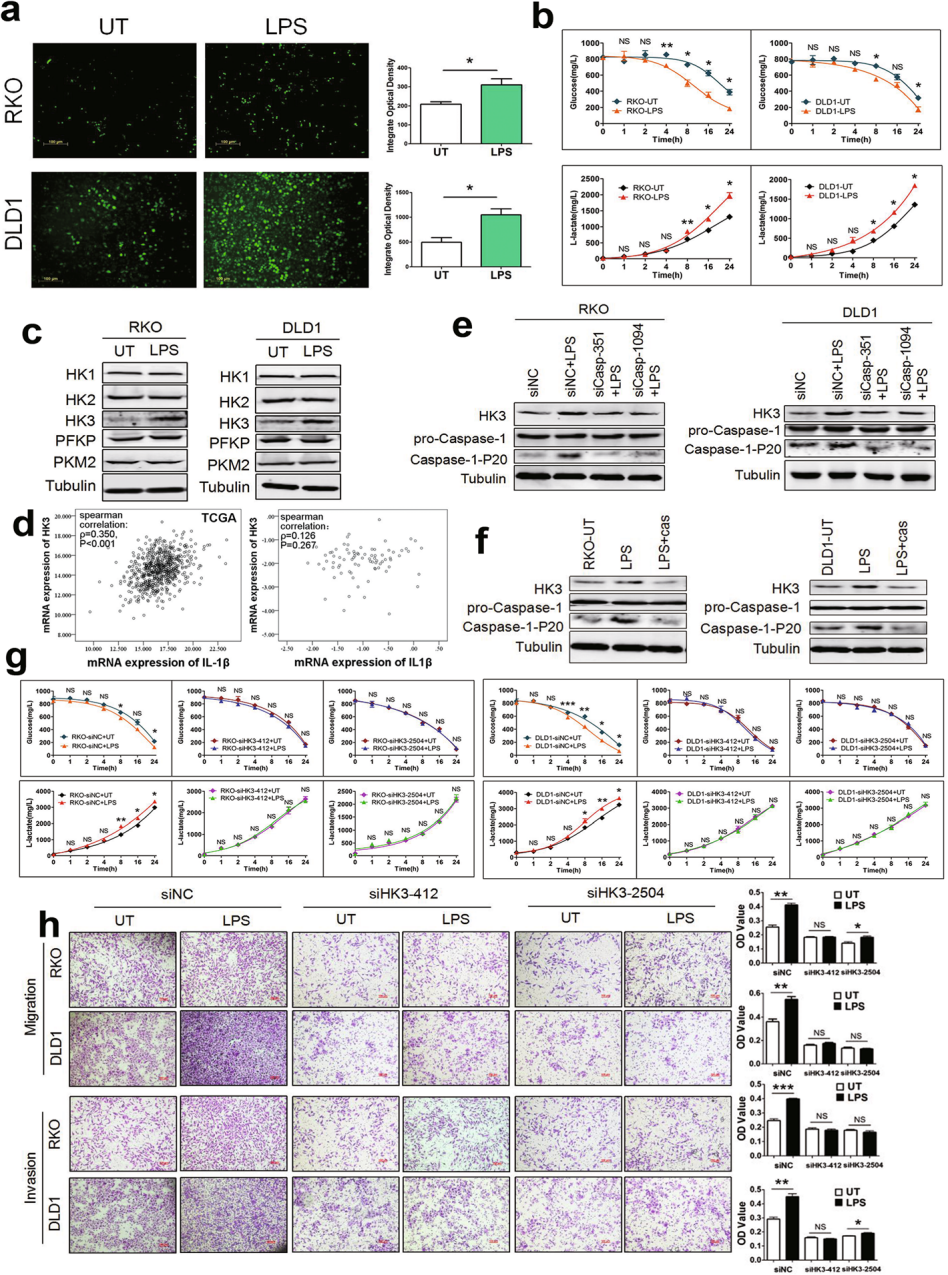
LPS enhances glycolysis and cell movements relying on HK3. **a** Effect of LPS on glucose uptake in RKO and DLD1 cells. Cells were cultured in glucose-free medium and treated with or without 1μg/ml LPS. After 24 h, cells were incubated with a fluorescent glucose derivative 2-NBDG for 30 min. Integrate optical density of fluorescence was detected using fluorescence microscopy and calculated by Image Pro Plus 6.0. Representative images from three independent experiments were shown. **b** Cells were cultured in glucose-free DMEM containing 10% FBS and 5mM glucose with or without LPS treatment. Cell medium supernatants were collected at 0, 1, 2, 4, 8, 16, and 24 h after LPS incubation. The concentration of glucose and lactate in cell medium supernatants was determined by glucose assay kit and lactic acid assay kit, respectively. **c** Cells were stimulated with 1μg/ml LPS for 24 h. Protein expression were determined by western blotting. Among several key glycolytic enzymes, only HK3 expression was obviously upregulated upon LPS treatment. **d** Scatter plot and spearman correlation analysis of HK3 and IL-1β mRNA expression levels in TCGA database (left) and our data (right) of CRC respectively. Logarithmic transformed data were used in our tissue samples. *ρ* was Spearman’s rank correlation coefficient. **e** Cells were transfected with non-targeting siRNA (siNC) or siRNA-Caspase-1 for 48 h, then treated with 1μg/ml LPS for 24 h. The effects of caspase-1 on HK3 expression with or without LPS treatment were examined by western blotting. **f** Cells were pretreated with 3μM caspase-1 inhibitor (Ac-YVAD-CHO) for 3 h, and then stimulated with LPS. Protein expression was determined by western blotting. **g** Cells that transient transfected with siRNA-HK3 or non-targeting siRNA (siNC) were treated with or without LPS. The initial glucose concentration was 5mM in culture medium. Glucose consumption and lactate production were detected by glucose assay kit and lactic acid assay kit in the indicated times respectively. **h** Transient siRNA transfection was performed by transfecting cells with 50nM control siRNA or siRNA-HK3 for 48 h, then treated with 1μg/ml LPS for 24 h. Transwell assay was used to detect cell migration and invasion ability; quantification of OD value was measured with microplate reader at 570 nm. Data are presented as mean±SEM. *P<0.05, **P<0.01, ***P<0.001

Next, we sought to identify whether LPS had effects on glucose metabolism of CRC cells. Cells with LPS treatment absorbed more 2-NBDG than those without LPS treatment, indicating that LPS enhanced glucose uptake (Fig. 4a). The glucose and lactate concentrations in cell culture supernatants were measured with commercial glucose and lactate assay kits, respectively. The results showed that LPS could promote glucose consumption and lactate production (Fig. 4b). Our observations suggest that metabolic reprogramming toward glycolysis via LPS increases the tumor metastatic potential of CRC cells.

Next, we detected the effects of LPS on the expression of glycolytic rate-limiting enzymes, including HK1, HK2, HK3, PFKP, and PKM2. The results showed that LPS markedly enhanced HK3 expression but had no significant effect on other enzymes (Fig. 4c). In addition, the correlation analysis showed that HK3 had a positive correlation with IL-1β in the TCGA database, but there was no significant correlation between HK3 and IL-1β in our data, which may be owing to availability of few samples in our laboratory (Fig. 4d). Further, the expression of caspase-1 was inhibited through siRNA or caspase-1 inhibitor Ac-YVAD-CHO to determine the relationship between inflammasome activation and HK3 expression. LPS did not upregulate HK3 expression after knockdown or inhibition of caspase-1 (Fig. 4e, f). These data suggest that LPS upregulates HK3 expression through inflammasome activation.

To further investigate the role of HK3 in LPS-upregulated glycolysis, we transiently transfected siRNA-HK3 and non-targeting siRNA (siNC) to cells. The results showed that LPS did not affect glycolysis after the knockdown of HK3, indicating that HK3 was essential to LPS-induced metabolic reprogramming (Fig. 4g). In addition, we found that LPS could promote the migration and invasion of CRC cells. However, the effect of LPS on cell motility was mitigated or disappeared after the knockdown of HK3, indicating that HK3 abundance was essential to LPS-induced migration and invasion (Fig. 4h). Moreover, there was no significant change in cell proliferation when HK3 was knockdown (Supplementary Fig. 2g, h).

### LPS regulates HK3 through NF-κB and snail

**Fig. 5:**
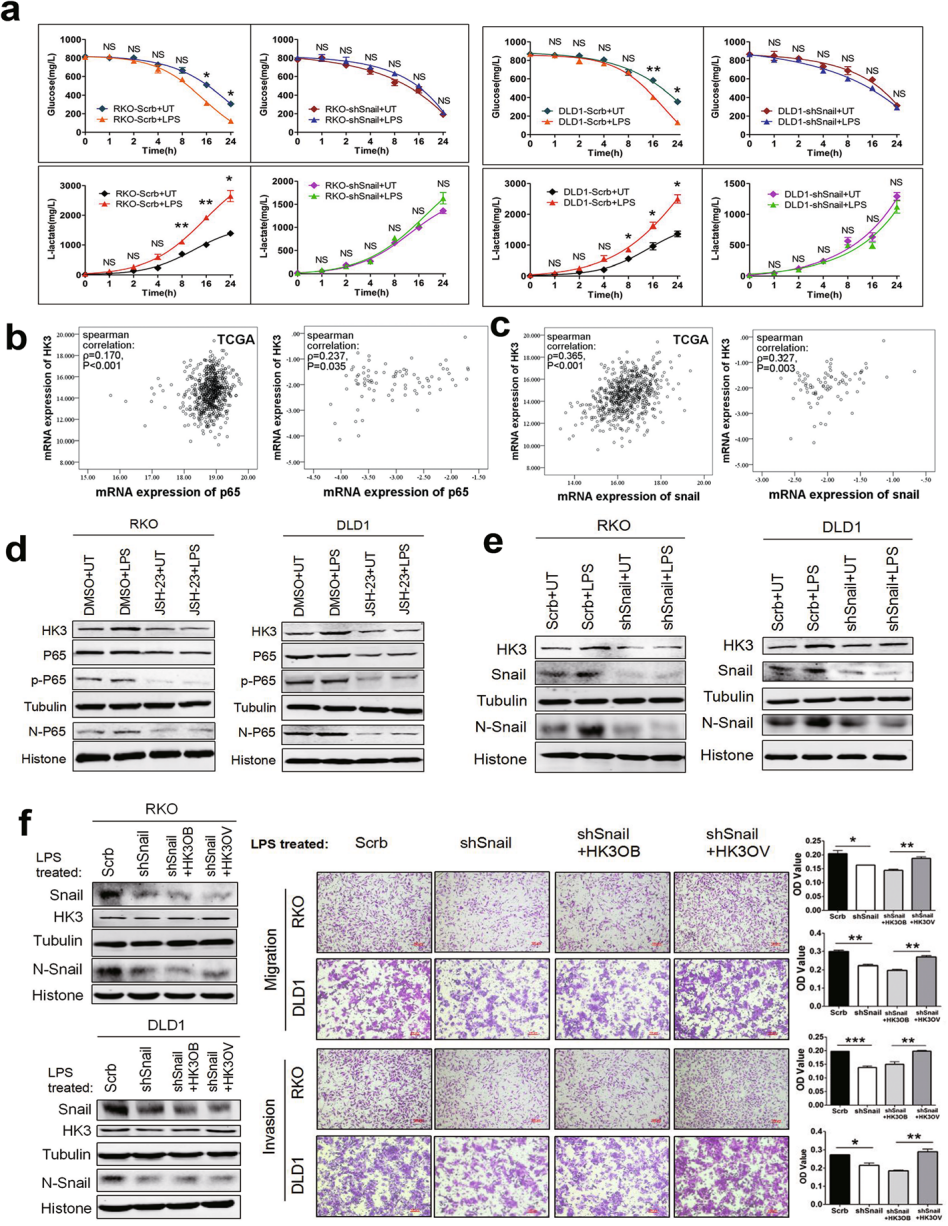
LPS-enhanced HK3 expression in a P65 and Snail-dependent manner in CRC cells. **a** Established cells that stably knockdown Snail were treated with or without 1μg/ml LPS. The initial glucose concentration was 5mM in culture medium. Glucose consumption and lactate production were detected by glucose assay kit and lactic acid assay kit in the indicated times. **b** Scatter plot and Spearman correlation analysis of p65 and HK3 mRNA expression levels in TCGA database (left) and our tissue samples (right) of CRC respectively. Logarithmic transformed data were used in our tissue samples. *ρ* was Spearman’s rank correlation coefficient. **c** Scatter plot and Spearman correlation analysis of snail and HK3 mRNA expression levels in TCGA database (left) and our tissue samples (right) of CRC respectively. Logarithmic transformed data were used in our tissue samples. *ρ* was Spearman’s rank correlation coefficient. **d** The effects of P65 on HK3 expression were examined by western blotting. Cells were pretreated with 50 μM NF-κB inhibitor JSH-23 for 1 h, followed by 1μg/ml LPS treatment for 24 h. **e** Snail knockdown cells (shSnail) were stimulated with or without LPS to examine the effect of Snail on LPS-upregulated HK3. Protein expression was detected by western blotting. **f** Snail knockdown cells (shSnail) were transfected with HK3 overexpression vector under LPS treatment. Transwell assay was used to detect cell migration and invasion. Quantification of OD value was measured with microplate reader at 570 nm. Data are presented as mean±SEM. *P<0.05, **P<0.01, ***P<0.001. N, nuclear; p-P65, phosphorylated P65

Recent studies have demonstrated that Snail is critically involved in glucose metabolism; however, the underlying mechanism is not fully elucidated. We measured glucose and lactate concentrations in Snail knockdown (shSnail) and control groups (Scrb) with or without LPS treatment. The results showed that LPS upregulated glucose consumption and lactate production, whereas knockdown of Snail significantly inhibited the enhancement of glycolysis induced by LPS (Fig. 5a).

LPS upregulated Snail expression and promoted Snail to enter nucleus through NF-κB. NF-κB and Snail are important transcription factors [10]. The correlation analysis in tumor tissues showed that HK3 positively related with P65 and Snail (Fig. 5b, c). Moreover, LPS could significantly upregulate HK3 expression, which could be reversed by NF-κB inhibitor JSH-23 (Fig. 5d). Interestingly, LPS did not upregulate HK3 expression after knockdown of Snail, indicating that Snail was also needed in LPS-induced HK3 expression (Fig. 5e). These results suggest that LPS fails to promote HK3 expression in cells depleted of either P65 or Snail, indicating that both proteins are essential for LPS-upregulated HK3 expression.

To further examine the effect of Snail/HK3 axis on cell migration and invasion upon LPS treatment, stable knockdown cell lines (shSnail) were transiently transfected with HK3 overexpression plasmid (HK3OV) or control vector (HK3OB). The overexpression of HK3 could rescue snail knockdown induced-cell movement inhibition upon LPS exposure (Fig. 5f) but did not affect cell proliferation (Supplementary Fig. 2i, j). These results imply that LPS promotes cell movements through the Snail/HK3 axis.

### NF-κB/snail forms a protein complex to upregulate HK3 promoter activity after LPS treatment

**Fig. 6:**
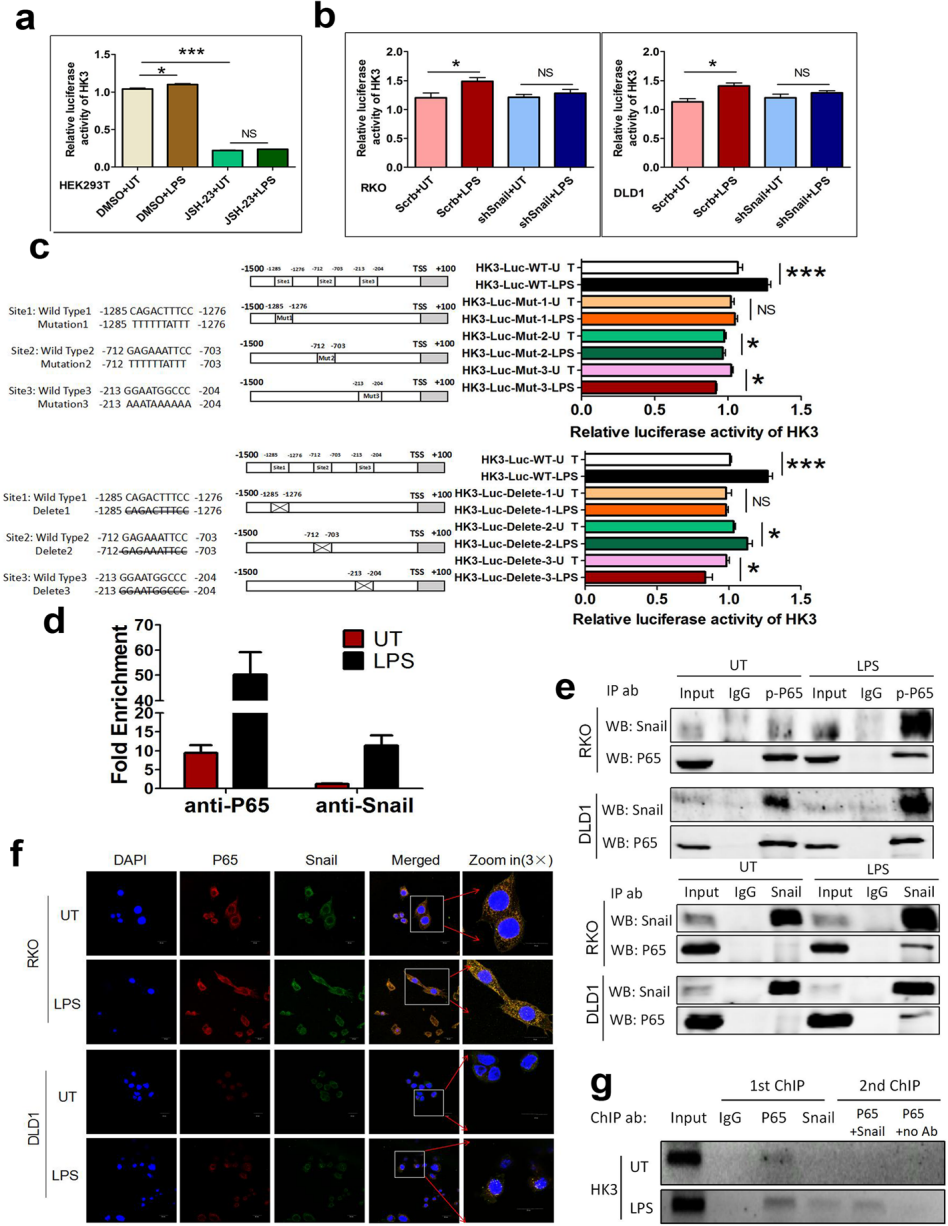
LPS promotes HK3 expression through inducing NF-κB/Snail protein complex formation and then binding to HK3 promoter region. **a** Quantification of the fold change of luciferase reporter assays of HK3 promoter activity. HEK239T cell was pretreated with DMSO or NF-κB inhibitor JSH-23, and then stimulated with LPS. Luciferase reporter gene plasmid containing the promoter region of HK3 was constructed (HK3-luc-WT). Cells were transfected with HK3-luc-WT plasmid DNA vector using LipoD293 transfection reagent. HK3 promoter activity was enhanced with LPS treatment, and NF-κB played an essential role during the process. **b** Quantification of the fold change of luciferase reporter assays of HK3 promoter in Snail-knockdown (shSnail) and control CRC cells (Scrb) with or without LPS treatment. HK3 promoter activity was enhanced with LPS treatment and Snail played an essential role during the process. **c** Luciferase reporter assays of HK3 promoter with mutant or deletion in predicted NF-κB binding site of HK3 promoter region. Luciferase reporter gene plasmid HK3-luc-WT contains the promoter region of HK3. A series of luciferase reporter gene plasmids encoding HK3 promoter region deletions (HK3-Luc-deletion) and mutants (HK3-Luc-mut) were constructed. All deletions and mutations of HK3-luc in the expression vector pGL3-basic were created using Mut Express II fast mutagenesis kit. **d** ChIP assay was carried out using ChIP assay kit to detect whether P65 or Snail could directly bind to HK3 promoter region. Combined HK3 DNA was quantified by real-time PCR. Fold enrichment was calculated relatively to the ChIP IgG control. **e** The protein interaction between P65 and Snail was confirmed by co-immunoprecipitation. Cells were treated with or without LPS, and cell lysates were prepared in IP lysis buffer. The indicated proteins were detected by western blotting. **f** The location of LPS-induced P65/Snail protein complex was confirmed by immunofluorescence. **g** ChIP-Re-ChIP assays were performed in LPS treated or untreated cells to examine whether P65 and Snail jointly regulate HK3 promoter activity. ChIP-Re-ChIP chromatin level was determined by PCR analysis. The PCR products were separated on a 1% agarose gel containing ethidium bromide for visualization and analysis. Data are presented as mean±SEM. *P<0.05, **P<0.01, ***P<0.001

Considering that NF-κB and Snail are essential in HK3 upregulation under LPS treatment, we further asked whether NF-κB and Snail could regulate HK3 promoter activity. We transfected luciferase reporter plasmid containing an HK3 promoter region to HEK293T cells. Dual luciferase reporter gene assay showed that HK3 promoter activity could be potentiated by LPS and blocked by NF-κB inhibitor JSH-23, suggesting that NF-κB plays an essential role in LPS-upregulated HK3 promoter activity (Fig. 6a). Next, we found that LPS significantly potentiated the relative luciferase activity of HK3 promoter, and Snail was needed in the process (Fig. 6b).

LPS failed to upregulate HK3 promoter activity in cells depleted of either NF-κB or Snail. Moreover, HK3 reporter activity was blocked under NF-κB inhibition but not under Snail knockdown conditions without LPS treatment. Presumably, NF-κB may bind to the HK3 promoter region and directly regulate the transcription of HK3 genes, and Snail assists this process upon LPS exposure. The JASPAR database predicted that there were three P65 binding sites (-1285/-1276, -712/-703 and -213/-204 from transcription start site) on the HK3 promoter region. Therefore, we constructed several deletions or mutants of the HK3 reporter gene based on the predicted binding sites. We found that LPS treatment significantly enhanced HK3 promoter activity in wild-type cells which expressed a full-length HK3 promoter (HK3-Luc-WT). However, when one of the three sites was deleted (HK3-Luc-deletion) or mutated (HK3-Luc-mut), LPS-induced HK3 promoter activity was suppressed or disappeared (Fig. 6c).

Next, we performed ChIP assay to further determine the mechanism of NF-κB and Snail-enhanced HK3 promoter activity upon LPS treatment. In untreated cells, approximately eight times more HK3 promoter chromatin was precipitated with the P65 antibody than with IgG antibody, but almost equal HK3 promoter chromatin was precipitated with the Snail antibody than with IgG antibody. Compared with the untreated group, HK3 promoter chromatin was significantly enriched by P65 or Snail antibody upon LPS treatment (Fig. 6d). These results indicate that P65 binds to the HK3 promoter with or without the existence of LPS. Notably, Snail only binds to the HK3 promoter under LPS treatment.

Based on the above results, we speculated that there might be protein interaction between P65 and Snail to jointly regulate HK3 transcription. The results of Co-IP verified that LPS could promote the formation of protein complex between P65 and Snail (Fig. 6e). Immunofluorescence revealed that LPS promoted the formation of protein complex between P65 and Snail in the nucleus (Fig. 6f). We next postulated that P65 and Snail binds to HK3 promoter in the form of a complex. ChIP-Re-ChIP assay demonstrated that the endogenous proteins P65 and Snail formed a complex, bind to the HK3 promoter region, and promote the transcription of HK3, thereby contributing to HK3 protein level upregulation upon LPS treatment (Fig. 6g).

### Metformin suppresses LPS-activated NF-κB/snail-HK3 axis to prevent glycolysis and metastasis

**Fig. 7:**
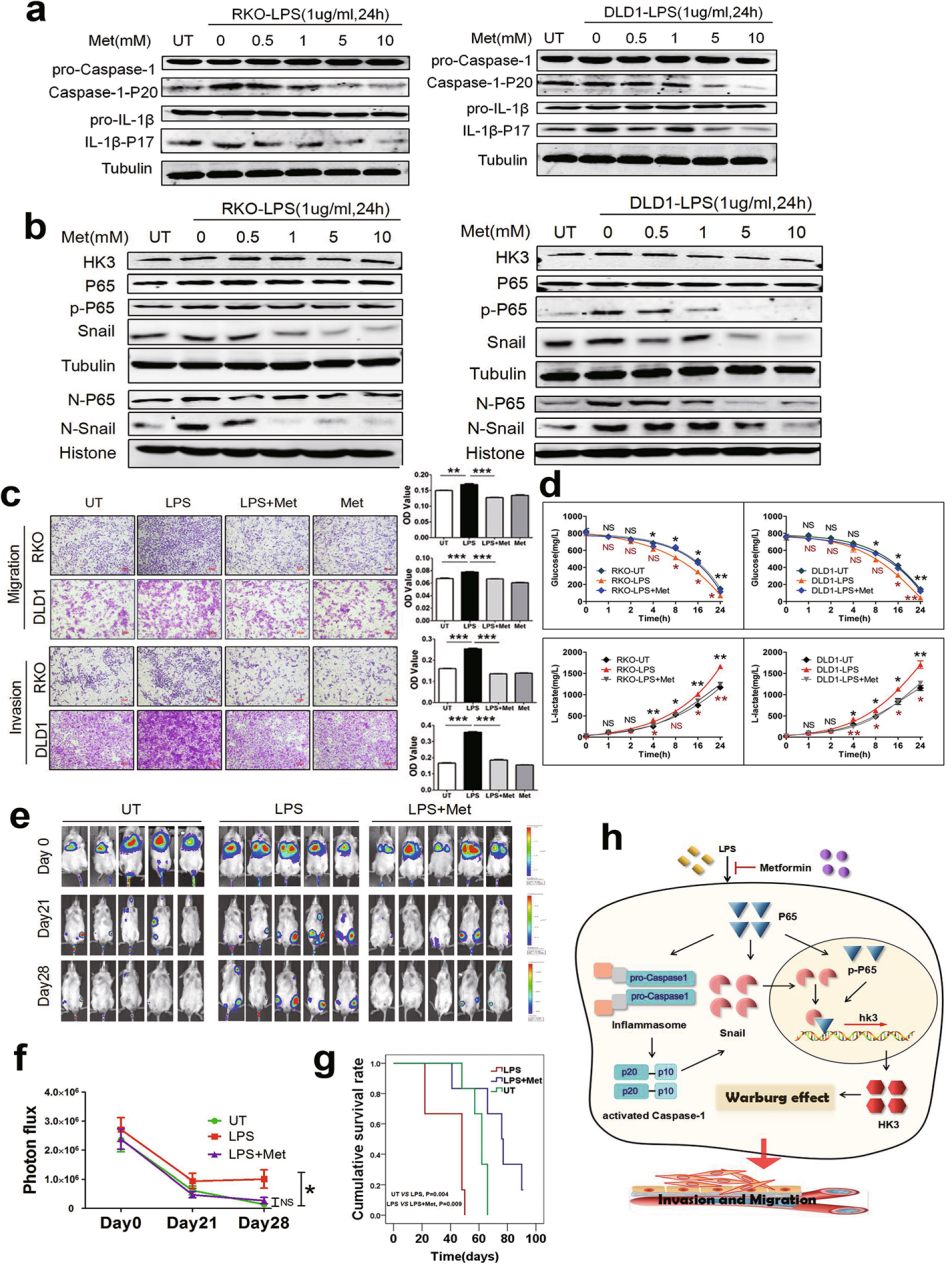
Metformin reverses the effect of LPS on promoting cell movements and glycolysis through NF-κB/Snail-HK3. **a** Cells were pretreated with metformin at 0, 0.5, 1, 5, and 10 mM for 1 h and then stimulated with 1μg/ml LPS for 24 h. Western blotting was used to detect the expression of inflammasome activation markers including Caspase-1-P20 and IL-1β-P17. **b** Metformin inhibited LPS-potentiated NF-κB, Snail, and HK3 expression. Cells were pretreated with 5mM metformin for 1 h, and then stimulated with LPS 1μg/ml for 24 hr. **c** LPS enhanced migration and invasion of CRC cells, which could be blocked by metformin. Cells were pretreated with 5 mM metformin for 1 h and then stimulated with 1μg/ml LPS for 24 h. OD values were measured by a microplate reader at 570 nm. **d** LPS upregulated glycolysis of CRC cells, which could be reversed by metformin. The initial glucose concentration was 5mM in culture medium. Cells were pretreated with 5 mM metformin for 1 h and then stimulated with 1μg/ml LPS for 24 h. Glucose consumption and lactate production were detected by glucose assay kit and lactic acid assay kit in the indicated times. **e**, **f** Images of luciferase signals (**e**) and quantification of photon flux (**f**) in metastatic luciferase foci in mice with tail-vein injection of untreated (UT), LPS treated, or LPS and metformin-treated RKO cells. **g** Survival curves of immunodeficient mice injected with untreated (UT), LPS treated, or LPS and metformin-treated RKO cells through tail-vein were plotted by Kaplan-Meier method and were compared by log-rank test. **h** Diagram of the effects of LPS on metastasis in CRC. LPS promotes metastasis through enhancing glycolysis via NF-κB/Snail/HK3 signaling axis, which could be reversed by metformin. Data are presented as mean±SEM. *P<0.05, **P<0.01, ***P<0.001. N, nuclear; p-P65, phosphorylated P65

Metformin, a widely used drug for diabetes, is reported to inhibit inflammasome activation [25]. We pretreated cells with metformin at 0, 0.5, 1, 5, and 10 mM for 1 h and then stimulated cells with LPS. Metformin pretreatment could inhibit LPS-induced inflammasome activation (Fig. 7a). Furthermore, metformin pretreatment could significantly prevent protein expression of NF-κB, Snail, and HK3 and nuclear translocation of NF-κB and Snail that were upregulated by LPS (Fig. 7b). Cells were stimulated with 5 mM metformin for 1 h in the following experiments. In addition, transwell assays and detection of glucose and lactate showed that metformin treatment could suppress LPS-induced cell motility and glycolysis enhancement (Fig. 7c, d). Metformin treatment had no effect on cell proliferation (Supplementary Fig. 2k, l). After tail vein injection of RKO cells with different treatments in mice, metastatic loci were observed in the whole body. Mice injected with LPS-treated cells had more metastasis than those injected with LPS-untreated cells, and metformin treatment completely reversed the pro-metastatic effect of LPS (Fig. 7e, f). Furthermore, survival analysis showed that metformin prolonged the survival time decreased by LPS (Fig. 7g). These data strongly suggest that metformin blocks the LPS-mediated NF-κB/Snail/HK3 axis, and consequently prevents LPS-promoted glycolysis and cell motility which prolongs survival time in CRC.

## Discussion

In our research, LPS activated inflammasomes in cancer cells to potentiate metastasis. In the presence of LPS, NF-κB-activated inflammasomes upregulated nuclear Snail expression and formed a protein complex with Snail, which bound to the promoter region of HK3 to enhance glycolysis, which could be inhibited by the antihyperglycemic agent metformin (Fig. 7h).

CRC is the leading cause of cancer-related death worldwide [25]. LPS is present at significantly higher concentrations in CRC tissues than in normal tissues. Previous studies on the tumor-promoting effect of LPS primarily focused on the immune microenvironment. Our results show that inflammasome activation in cancer cell also plays a vital role during LPS-induced metastasis. Inhibiting inflammasome activity can ameliorate the migration and invasion induced by LPS. Inflammasomes are classified as NLR, AIM2, and IFI16 according to sensor molecules; however, we did not study which of these functioned during LPS treatment in CRC cells, and this should be investigated in future studies.

LPS activates Toll-like receptor 4 (TLR4) in cell membrane or cytoplasm and primes inflammasome activation mediated by NF-κB, which occurs not only in macrophages but also in colonic epithelial cells [6]. NF-κB suggests a mechanistic link between inflammation and cancer, which plays central roles in both innate immunity and tumor progression. In keratinocytes, P65 directly binds to the motif of the caspase-1 promoter to initiate its expression [26]. In our experiments, knockdown of P65 and inhibition of NF-κB completely suppress caspase-1 cleavage and IL-1β maturation, indicating that LPS activates caspase-1 depending on NF-κB. Concurrently, LPS does not promote migration and invasion after repression of NF-κB.

Snail is a critical molecule in inflammatory cytokine-induced metastasis, and its stabilization depends on NF-κB [10]. We found that the expression of total and nuclear Snail is upregulated under LPS treatment, which could be blocked by inhibitor of caspase-1 or knockdown of P65. LPS failed to promote metastasis when Snail was knocked down. Cumulatively, NF-κB and Snail both play important roles in LPS-induced metastasis. Snail can also regulate aerobic glycolysis. For example, Snail suppresses PFKP to switch the glucose flux toward the pentose phosphate pathway, generating NADPH to facilitate the survival of cancer cells under oxidative stress conditions [11]. Snail downregulates glycolysis induction by FBP1 and increases glucose uptake [12]. We found that Snail plays a critical role in LPS-induced aerobic glycolysis.

Aerobic glycolysis, also known as the Warburg effect, generates ATP, provides glycolytic intermediates, and alleviates oxidative stress. This facilitates survival of cancer cells during the stringent metastatic process. There is a dynamic interface between glycolysis and inflammasome activation. In macrophages, LPS enhances glycolysis through NLRP3/IL1β [15]. Glycolytic enzymes, such as HK1 and PKM2, also induce inflammasome activation [27]. Our results reveal that LPS-potentiated cancer cell motility depends on increased glucose uptake and glycolysis. Among the rate-limiting enzymes in glycolysis, HK3 (the first enzyme in glycolysis which converts glucose to glucose-6-phosphate) was significantly upregulated under LPS treatment.

Compared with the three hexokinase isozymes, HK1, HK2, and HK4, the role and mechanism of action of HK3 in cancer is less reported. Our study shows that it is HK3 rather than other rate-limiting enzymes in glycolysis that increases after LPS treatment. Pudova et al. reported that the expression of HK3 is higher in CRC tissues than in matched normal tissues, probably owing to metastasis [16]. HK3 protects cells from oxidant-induced death and increases ATP production under conditions of hypoxia [28]. Our research reveals that HK3 plays an important role in regulating glycolysis in intestinal microbial-induced metastasis of CRC.

Recent evidence suggests that metformin, widely known as a drug for the treatment of type 2 diabetes, plays important roles in several cellular processes [29–31]. Metformin can block tumor progression induced by inflammasome activation in tumor-infiltrating immune cells, protect the intestinal barrier from the LPS-triggered damage by alleviating NF-κB phosphorylation, and directly inhibit the enzymatic activity of HK1 and HK2 [17, 18, 32]. Here, we prove that metformin attenuates caspase-1 activation and the NF-κB/Snail/HK3 signaling axis and inhibits LPS-promoted glycolysis and metastasis.

Compared with a healthy person, caspase-1 cleavage and IL-1β maturation are elevated in patients with type 2 diabetes mellitus (T2DM), which can be significantly suppressed by metformin [19]. In a meta-analysis, patients with diabetes have an 18% lower 5-year survival rate in CRC [33]. Intriguingly, metformin improves the overall and cancer-specific survival rates of patients with CRC and diabetes [34]. Nevertheless, there are conflicting results regarding the effects of metformin on the incidence and prognosis of cancer [21, 35, 36]. However, in these studies, the diversity of the microbiota was neglected. Our findings suggest that metformin attenuates LPS-induced metastasis and improves the prognosis and survival of mice with LPS treatment.

## Conclusions

In conclusion, LPS activates inflammasomes in cancer cells, and inflammasome activation is involved in reinforcing cell motility and glycolysis. LPS promotes metastasis through boosting glycolysis via the NF-κB/Snail/HK3 signaling axis, and this effect could be repressed by metformin. Drugs targeting caspase-1 activation and adjustment of the composition of intestinal flora can be introduced in CRC treatments.

## Supplementary Information

**Additional file 1: Supplementary file1**: Supplementary Materials and Methods.

**Additional file 2: Supplementary Fig. 1**. LPS promotes inflammasome activation of RKO cells, but does not influence proliferation and cell death of CRC cells. a. RKO cells were treated with 0,1,2,4,8 μg/ml LPS for 24 hrs. Protein expression of inflammasome activation markers Caspase-1-P20 (activated Caspase-1) and IL-1β-P17 (matured IL-1β) were measured by western blotting. b. RKO cells were treated with 0,1,2,4,8 μg/ml LPS for 24 hrs. Quantification of Caspase-1 enzyme activity was performed with Caspase-Glo^®^ 1 Inflammasome Assay Kit. c. RKO cells were exposed to 1μg/ml LPS for increasing times (0, 4, 8, 12, 24, 48 hrs). Protein expression of inflammasome activation markers including Caspase-1-P20 and IL-1β-P17 were measured by western blotting. d. Cell proliferation was detected using the CCK8 assay (Beyotime, C0038). The cell proliferation curves were plotted by measuring 450nm absorbance at indicated time point (0, 1, 2, 3, 4 and 5 days). Experiments were performed in triplicate. e. Cells apoptosis was examined by flow cytometry using the Annexin V-FITC/PI apoptosis kit (MultiScience, Cat# AP101). Early apoptosis was assigned as the B4 quadrant and late apoptosis was assigned as the B2 quadrant. The sum of percentage of B4 and B2 was adopted in statistical analysis. Data were shown as mean± SEM. NS, no significantly difference.

**Additional file 3: Supplementary Fig. 2**. The effect of different treatment on cell proliferation. Cell proliferation was detected using the CCK8 assay (Beyotime, C0038). The OD values were measured at 450nm on indicated time point (1, 2, 3 and 4 days). a, b. Both LPS and Ac-YVAD-CHO(cas) had no effect on cell proliferation. c, d. There was no significant difference in cell proliferation when P65 was transient knockdown. e, f. Established or transient knockdown of Snail had no effect on cell proliferation. g, h. There was no significant difference in cell proliferation when HK3 was transient knockdown. i, j. Under the condition of Snail knockdown, overexpression of HK3 did not affect cell proliferation. k, l. Both LPS and Metformin had no effect on cell proliferation. Experiments were performed in triplicate. Data were shown as mean± SEM. NS, no significantly difference.

**Additional file 4: Supplementary Fig. 3**. LPS promoted migration and invasion depending on glucose concentration. a. The effect of different glucose concentration on cell migration. Migration was determined by transwell assay. Cells were cultured with the indicated glucose concentrations (0, 5, 10, 15mM) for 24 hrs and stimulated with or without 1μg/ml LPS. b. The effect of different glucose concentration on cell invasion. Invasion was determined by transwell assay. Cells were cultured with the indicated glucose concentrations (0, 5, 10, 15mM) for 24 hrs and stimulated with or without 1μg/ml LPS.

## Data Availability

Not applicable.

## Abbreviations

CRC: Colorectal cancer
FBS: Fetal Bovine Serum
LPS: Lipopolysaccharide
ChIP: Chromatin immunoprecipitation
Co-IP: Co-immunoprecipitation
HK: Hexokinase
NF-κB: Nuclear factor-κB
TLR4: Toll-like receptor 4
PFKP: Phosphofructokinase, platelet
PKM2: Pyruvate Kinase M2
FBP1: Fructose-1,6-biphosphatase
PFKFB3: 6-Phosphofructo-2-kinase/fructose-2,6-bisphosphatase 3
CCK8: Cell Counting Kit-8
2-NBDG: 2-(N-(7-Nitrobenz-2-oxa-1,3-diazol-4-yl)Amino)-2-Deoxyglucose
PBS: Phosphate buffer solution
DAPI: 4’,6-Diamidino-2-phenylindole
H&E: Hematoxylin and eosin
