# Supplementary material for: Lipopolysaccharide promotes metastasis via acceleration of glycolysis by the nuclear factor-κB/snail/hexokinase3 signaling axis in colorectal cancer
Source: Cancer Metab. 2021 May 12;9:23. doi: 10.1186/s40170-021-00260-x (PMC8117511; doi:10.1186/s40170-021-00260-x)
Supplement: Supplementary file 1 — Additional file 1: Supplementary file1: Supplementary Materials and Methods. [file 40170_2021_260_MOESM1_ESM.docx]

**Supplementary Materials and Methods**

**RNA interference**

To knockdown p65, snail and hk3, cells were seeded in 6-well plates at the density of 2×10^5^ cells per well and incubated overnight. Next day, cells were transfected with p65 siRNA, snail siRNA, hk3 siRNA or non-targeting siRNA (all from GenePharma, Shanghai, China, final concentration 50 nM) using GenMute siRNA Transfection Reagent (SignaGen Laboratories, MD, USA). Gene knockdown efficacy was determined by western blotting or Real-time PCR. The siRNA sequences are listed in supplementary Table 1.

**Supplementary Table 1**

| **Clone name** | **Target Sequence** |
| --- | --- |
| siNC-sense | 5’-UUCUCCGAACGUGUCACGUTT-3’ |
| siNC-antisense | 5’-ACGUGACACGUUCGGAGAATT-3’ |
| siP65-2-sense | 5’-GAUCAAUGGCUACACAGGATT-3’ |
| siP65-2-antisense | 5’-UCCUGUGUAGCCAUUGAUCTT-3’ |
| siP65-289-sense | 5’-GGAGCACAGAUACCACCAATT-3’ |
| siP65-289-antisense | 5’-UUGGUGGUAUCUGUGCUCCTT-3’ |
| siSnail-sense | 5’-GCCUUCAACUGCAAAUACUTT-3’ |
| siSnail-antisense | 5’-AGUAUUUGCAGUUGAAGGCTT-3’ |
| siHK3-412-sense | 5’-GGGUGACUCUAACUGGCAUTT-3’ |
| siHK3-412-antisense | 5’-AUGCCAGUUAGAGUCACCCTT-3’ |
| siHK3-2504-sense | 5’-GCAGGUCCGAGCCAUCCUATT-3’ |
| siHK3-2504-antisense | 5’-UAGGAUGGCUCGGACCUGCTT-3’ |

**Real-time PCR**

Total RNAs from patient tissues were isolated using the Trizol (Pufei, Shanghai, China). Then quantitative real-time PCR was performed using TB Green PCR master mix (Takara, Tokyo, Japan) on a LightCycler480 Real Time PCR Detection System (Roche, Basel, Switzerland). The relative expression level was calculated using the 2^−ΔCt^ method and normalized by tubulin expression. A negative control (nuclease-free water) was included throughout the experiments to detect contamination and to determine the degree of dimer formation. All real-time PCR reactions were carried out in triplicates. The sequences of the primers used are provided in supplementary Table 2.

**Supplementary Table 2**

| **Primer** | **Sequence** |
| --- | --- |
| IL-1β-F | 5′-GCAGAAGTACCTGAGCTCGC-3’ |
| IL-1β-R | 5′-CATGGCCACAACAACTGACG-3’ |
| P65-F | 5′-GGGCATGCGCTTCCGCTAC-3’ |
| P65-R | 5′-TCCCCACGCTGCTCTTCTTGGA-3’ |
| Snail-F | 5′-CTAGGCCCTGGCTGCTACAAG-3’ |
| Snail-R | 5′-AGCGGGGACATCCTGAGCA-3’ |
| Tubulin-F | 5′-GGGACCATGGACTCTGTTCG-3’ |
| Tubulin-R | 5′-CATGGTGGCTGAGACAAGGT-3’ |

HK3 primers were purchased from Sino Biological lnc. (Beijing, China)

**Western blotting**

Tumor tissues and in vitro cultured cells were lysed with RIPA lysis buffer (Beyotime, Shanghai, China) containing protease inhibitor mixture PMSF (Beyotime, Shanghai, China) and phosphatase inhibitor cocktail (MCE, NJ, USA). The nuclear protein was extracted using a Nuclear and Cytoplasmic Protein Extraction Kit (Keygen Biotech, Nanjing, China). For supernatant protein, cell culture mediums were concentrated by centrifugation at 5000 rpm with Amicon Ultra-15 Centrifugal Filter Devices -3K (Millipore, MA, USA) for 2 hr according to the manufacturer’s protocol.

Subsequently, equal amount of protein extracts was loaded onto polyacrylamide gels and separated by SDS-PAGE and then transferred on nitrocellulose membranes. GAPDH or tubulin was used as a loading control for western blotting. Immunocomplexes were detected by Odyssey Imager (LI-COR, NE, USA). Results were confirmed by at least three independent experiments. The information of primary antibodies of western blotting was provided in supplementary Table 3.

**Supplementary Table 3**

| **Antibody** | **Dilution** | **Manufacturer** |
| --- | --- | --- |
| Pro-Caspase-1 | 1:1000 | Abcam（ab179515） |
| Actived Caspase-1(p20) | 1:500 | Bioworld（BS61788） |
| Pro-IL-1β | 1:1000 | CST（12703） |
| IL-1β-P17 | 1:1000 | CST（12242） |
| NF-κB p65NF-κB | 1:1000 | CST（4764） |
| NF-κB p65Phospho-NF-κB | 1:1000 | CST（3033） |
| Snail | 1:1000 | CST（3879） |
| Hexokinase1 | 1:1000 | Abcam（ab110529） |
| Hexokinase2 | 1:1000 | Abcam（ab104836） |
| Hexokinase3 | 1:100 | Santa Cruz（sc74488） |
| PFKP | 1:1000 | Abcam（ab204131） |
| PKM2 | 1:100 | Santa Cruz（sc100538） |

**Plasmids construction and Dual-Luciferase reporter assay**

Lentivirus vector plasmids expressing scrambled sequences (gcttcgcgccgtagtctta, Cat# CSHCTR001-LVRU6GP) or shRNA sequences (gcaacaaggaatacctcagcc, Cat#HSH017573-LVRU6GP) targeting human snail were purchased from GeneCopoeia (Nanjing, China). Stable DLD-1-shSnail, RKO-shSnail cell lines and their controls were generated by lentiviral transduction and selected by flow cytometry sorting. Knockdown efficiency of the target proteins was evaluated by western blotting (WB).

HK3 promoter sequence data were retrieved from the Eukaryotic Promoter Database (https://epd.epfl.ch//index.php). The HK3 promoter region (−1500 to +100) was amplified from human genomic DNA by using a Prime STAR HS DNA Polymerase with GC buffer kit (Takara, Tokyo, Japan). The primers to amplify the HK3 promoter region are listed in below:

HK3 promoter-F:

5’-CGTGCTAGCCCGGGCTCGAGAGCCAGGCTGTCCCAGCT-3’

HK3 promoter-R:

5’-AGTACCGGAATGCCAAGCTTGGCTGGAGTGAGCCCTGACT-3’

A HK3 promoter luciferase reporter vector(HK3-luc-WT) was prepared by ligating HK3 promoter region to pGL3-basic firefly luciferase reporter gene vector using the restriction enzymes XhoI and HindIII sites.

The transcription factor binding sites (TFBS) of P65 in HK3 promoter region was predicted using JASPAR database (http://jaspar.genereg.net/). All deletions (HK3-Luc-delete) and mutations (HK3-Luc-Mut) in the pGL3-basic HK3-luc-WT were created by Mut Express II fast mutagenesis kit V2 (Vazyme Biotech, Nanjing, China). Plasmid transfection was performed using Lipofectamine 2000 Reagent (Invitrogen, CA, USA) according to the manufacturer’s instructions. The sequences of the primer used are provided in Supplementary Table 4.

**Supplementary Table 4**

| **Primer** | **Sequence** |
| --- | --- |
| HK3-Luc-delete-1-F | 5’-TTGACCCTGGCATTCTGACTAGGGAACCAGGAC-3’ |
| HK3-Luc-delete-1-R | 5’-TCAGAATGCCAGGGTCAAGGCCAGTGGAGGGAG-3’ |
| HK3-Luc-delete-2-F | 5’-GTGCCTTTGAGCACAGAAAAACAGCTTGTTCGC-3’ |
| HK3-Luc-delete-2-R | 5’-TTCTGTGCTCAAAGGCACAGAACCTTGGGGCAC-3’ |
| HK3-Luc-delete-3-F | 5’-GCATTGTAGTAGACCCAGTTCAACGCTCCACTT-3’ |
| HK3-Luc-delete-3-R | 5’-CTGGGTCTACTACAATGCTGAGGGCTGTTTATT-3’ |
| HK3-Luc-Mut-1-F | 5’-GACCTTTTTTATTTCTGGCATTCTGACTAGGGAACC-3’ |
| HK3-Luc-Mut-1-R | 5’-CCAGAAATAAAAAAGGTCAAGGCCAGTGGAGGG-3’ |
| HK3-Luc-Mut-2-F | 5’-GAGTTTTTTATTTCACAGAAAAACAGCTTGTTCGC-3’ |
| HK3-Luc-Mut-2-R | 5’-CTGTGAAATAAAAAACTCAAAGGCACAGAACCTTGGG-3’ |
| HK3-Luc-Mut-3-F | 5’-GTAGAGAAATAAAAAAAAAATCTTAGACCCAGTTCAACGC-3’ |
| HK3-Luc-Mut-3-R | 5’-TTTTTTTTATTTCTCTACAATGCTGAGGGCTGTTT-3’ |

The plasmid of pWZL Neo Myr Flag HK3 was a gift from William Hahn & Jean Zhao (Addgene plasmid # 20414; http://n2t.net/addgene:20414; RRID:Addgene_20414). The plasmid of pWZL-Neo-Myr-Flag-DEST was a gift from Jean Zhao (Addgene plasmid # 15300; http://n2t.net/addgene:15300 ; RRID:Addgene_15300).

**Chromatin immunoprecipitation (ChIP) assay**

ChIP was performed with the ChIP Express Kit (Active Motif, CA, USA) according to the manufacturer’s protocol. Briefly, cross-linked cells were scraped off and collected by centrifugation. Shearing was performed by sonication in a Diagenode Bioruptor to obtain 200–1500 bp DNA fragments. Input samples consisted of 10 μl sonicated chromatin was removed before the IP. For each ChIP, equal amounts of sheared chromatin were incubated overnight at 4℃ with anti-P65 (CST, MA, USA) or anti Snail (R&D, MN, USA) antibody, protein G magnetic beads and protease inhibitors. Then, bound DNA-protein complexes were eluted and cross-links were reversed. After adding Proteinase K Stop Solution, DNA samples were subsequently used as templates and analyzed by quantitative real-time PCR. Serial dilutions of input material were used to create standard curves for each individual primer pair. Fold enrichment was calculated over the ChIP IgG control (fold enrichment >5 was designated as significant). The input DNA was used to make a standard curve to calculate fold enrichments. The sequences of the primer used are provided in supplementary Table 5.

**Supplementary Table 5**

| **Primer** | **Sequence** |
| --- | --- |
| HK3-site1-F | 5’-GCCATCCTTCTCCCTCCAC-3’ |
| HK3-site1-R | 5’-GGGCTCTTCATCTGCGTTTC-3’ |
| HK3-site2-F | 5’-TTGAAGGCATGGATACACTTT-3’ |
| HK3-site2-R | 5’-CAGCAGGACTGATGTTTGG-3’ |
| HK3-site3-F | 5’-GTGCTCCCACATTGCCTCTT-3’ |
| HK3-site3-R | 5’-CCTTCCAAGCCTGCCCTAA-3’ |

**Cell migration and invasion assay**

Transwell assay was performed using chambers with membranes of 8 μm pore size (Corning, NY, USA). For migration assay, the lower side of the filter was coated with fibronectin. For invasion assay, the lower side of the filter was coated with fibronectin and the upper side of the filter was covered with matrigel. The upper compartments were seeded with 1 × 10^5^ DLD-1 or RKO cells in RPMI 1640 without serum, and RPMI 1640 containing 10% FBS as a chemoattractant was added to the lower compartments of 24-well plates. After a 48 hr incubation period, non-invading cells were removed from the upper surface of the membrane by scrubbing with cotton-tipped swabs and those on the lower surface were fixed in 4% paraformaldehyde for 10 min and then stained with 0.5% crystal violet (Beyotime, Shanghai, China) for 10 min. Images of cells on the lower face of the filters were captured in fields at 100× magnification using a digital microscopy. Cells were then digested in 33% acetic acid to dissolve the crystal violet absorbed by cells on the lower face of the filter, and then quantified by measuring absorbance at 570 nm with a 96-well plate on a microplate reader (Molecular Devices, CA, USA). All experiments were performed in triplicate.

**CCK8 assay**

Cell proliferation was determined using Cell Counting Kit-8 (CCK8) (Beyotime, C0038). Briefly, 2× 10^3^cells were seeded into 96-well plates, and fresh culture medium with 10μl CCK8 [solution](https://www.sciencedirect.com/topics/immunology-and-microbiology/solution-and-solubility) was added at 0, 24, 48, 72, 96 hours and incubated for 3 hrs at 37°C.The absorbance was measured at 450nm by a microplate reader (Molecular Devices, M5). All measurements were performed in triplicate.

**Flow cytometry analysis**

Apoptosis assay was performed using Annexin V-FITC/PI apoptosis kit (MultiScience, Cat# AP101) according to the manufacturer's instructions. Briefly, cells were harvested using trypsin, washed twice with ice-cold PBS and resuspended in 1×Binding Buffer at a concentration of 1×10^6^cells/ml. Solution was transferred into a culture tube, then 10μl PI and 5μl FITC Annexin V were added. After incubated for 5 min at room temperature in the dark, cell death was analyzed by flow cytometry. Data acquisition and analysis were performed with Cytomic FC 500MCL.
