# Supplementary figures and images for: Lipopolysaccharide promotes metastasis via acceleration of glycolysis by the nuclear factor-κB/snail/hexokinase3 signaling axis in colorectal cancer
Source: Cancer Metab. 2021 May 12;9:23. doi: 10.1186/s40170-021-00260-x (PMC8117511; doi:10.1186/s40170-021-00260-x)

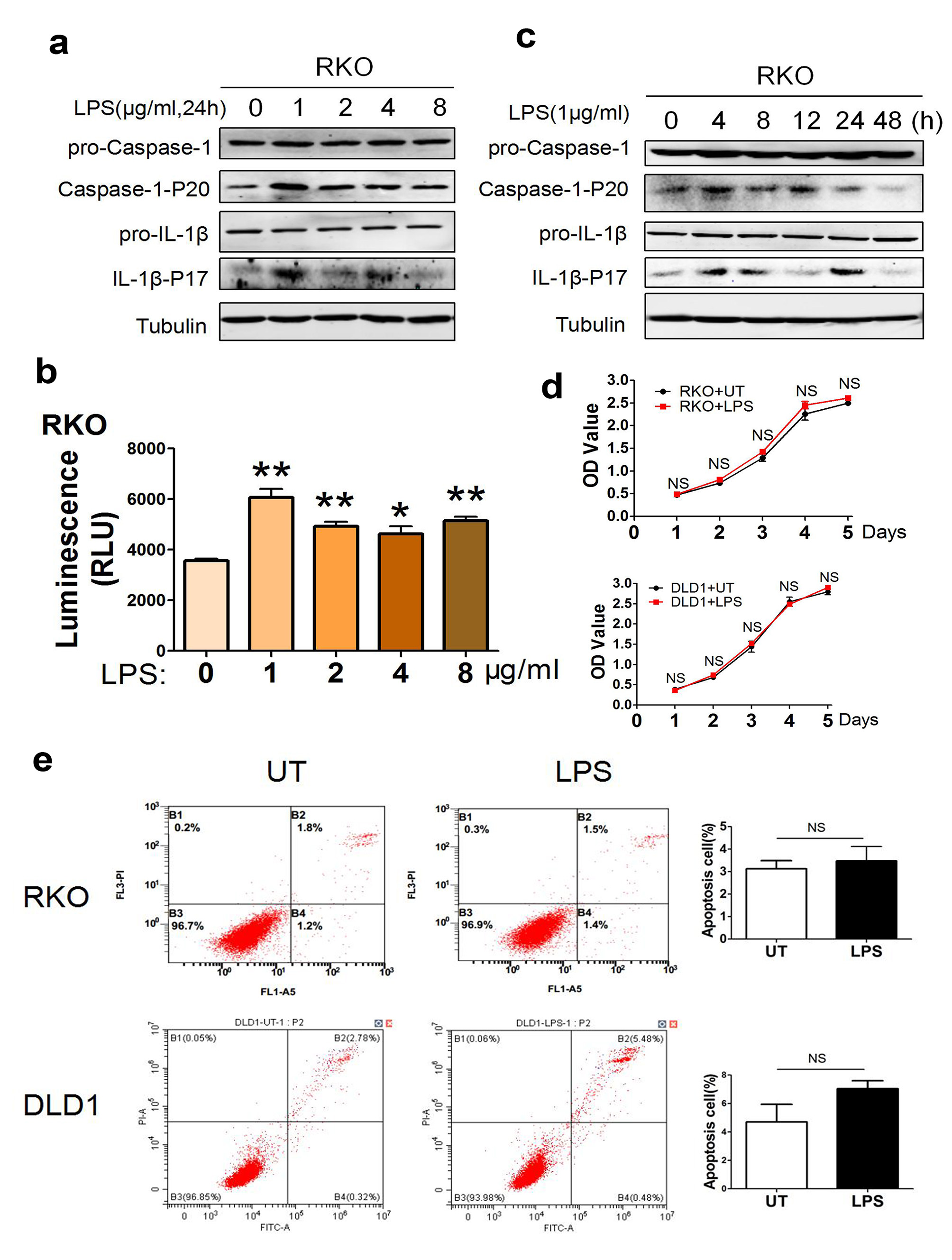

Supplement: Supplementary file 2 — Additional file 2: Supplementary Fig. 1. LPS promotes inflammasome activation of RKO cells, but does not influence proliferation and cell death of CRC cells. a. RKO cells were treated with 0,1,2,4,8 μg/ml LPS for 24 hrs. Protein expression of inflammasome activation markers Caspase-1-P20 (activated Caspase-1) and IL-1β-P17 (matured IL-1β) were measured by western blotting. b. RKO cells were treated with 0,1,2,4,8 μg/ml LPS for 24 hrs. Quantification of Caspase-1 enzyme activity was performed with Caspase-Glo® 1 Inflammasome Assay Kit. c. RKO cells were exposed to 1μg/ml LPS for increasing times (0, 4, 8, 12, 24, 48 hrs). Protein expression of inflammasome activation markers including Caspase-1-P20 and IL-1β-P17 were measured by western blotting. d. Cell proliferation was detected using the CCK8 assay (Beyotime, C0038). The cell proliferation curves were plotted by measuring 450nm absorbance at indicated time point (0, 1, 2, 3, 4 and 5 days). Experiments were performed in triplicate. e. Cells apoptosis was examined by flow cytometry using the Annexin V-FITC/PI apoptosis kit (MultiScience, Cat# AP101). Early apoptosis was assigned as the B4 quadrant and late apoptosis was assigned as the B2 quadrant. The sum of percentage of B4 and B2 was adopted in statistical analysis. Data were shown as mean± SEM. NS, no significantly difference. [file 40170_2021_260_MOESM2_ESM.jpg]

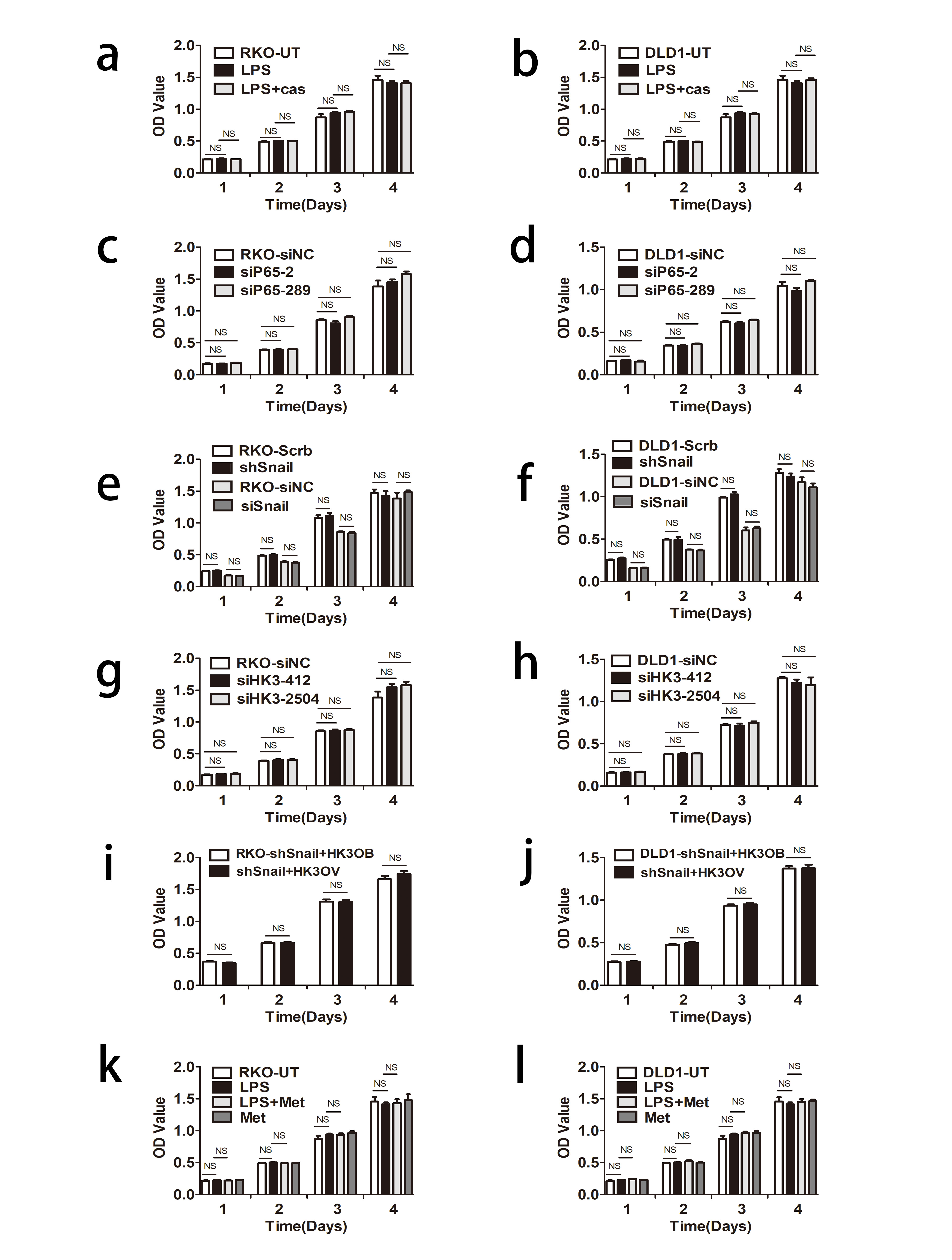

Supplement: Supplementary file 3 — Additional file 3: Supplementary Fig. 2. The effect of different treatment on cell proliferation. Cell proliferation was detected using the CCK8 assay (Beyotime, C0038). The OD values were measured at 450nm on indicated time point (1, 2, 3 and 4 days). a, b. Both LPS and Ac-YVAD-CHO(cas) had no effect on cell proliferation. c, d. There was no significant difference in cell proliferation when P65 was transient knockdown. e, f. Established or transient knockdown of Snail had no effect on cell proliferation. g, h. There was no significant difference in cell proliferation when HK3 was transient knockdown. i, j. Under the condition of Snail knockdown, overexpression of HK3 did not affect cell proliferation. k, l. Both LPS and Metformin had no effect on cell proliferation. Experiments were performed in triplicate. Data were shown as mean± SEM. NS, no significantly difference. [file 40170_2021_260_MOESM3_ESM.jpg]

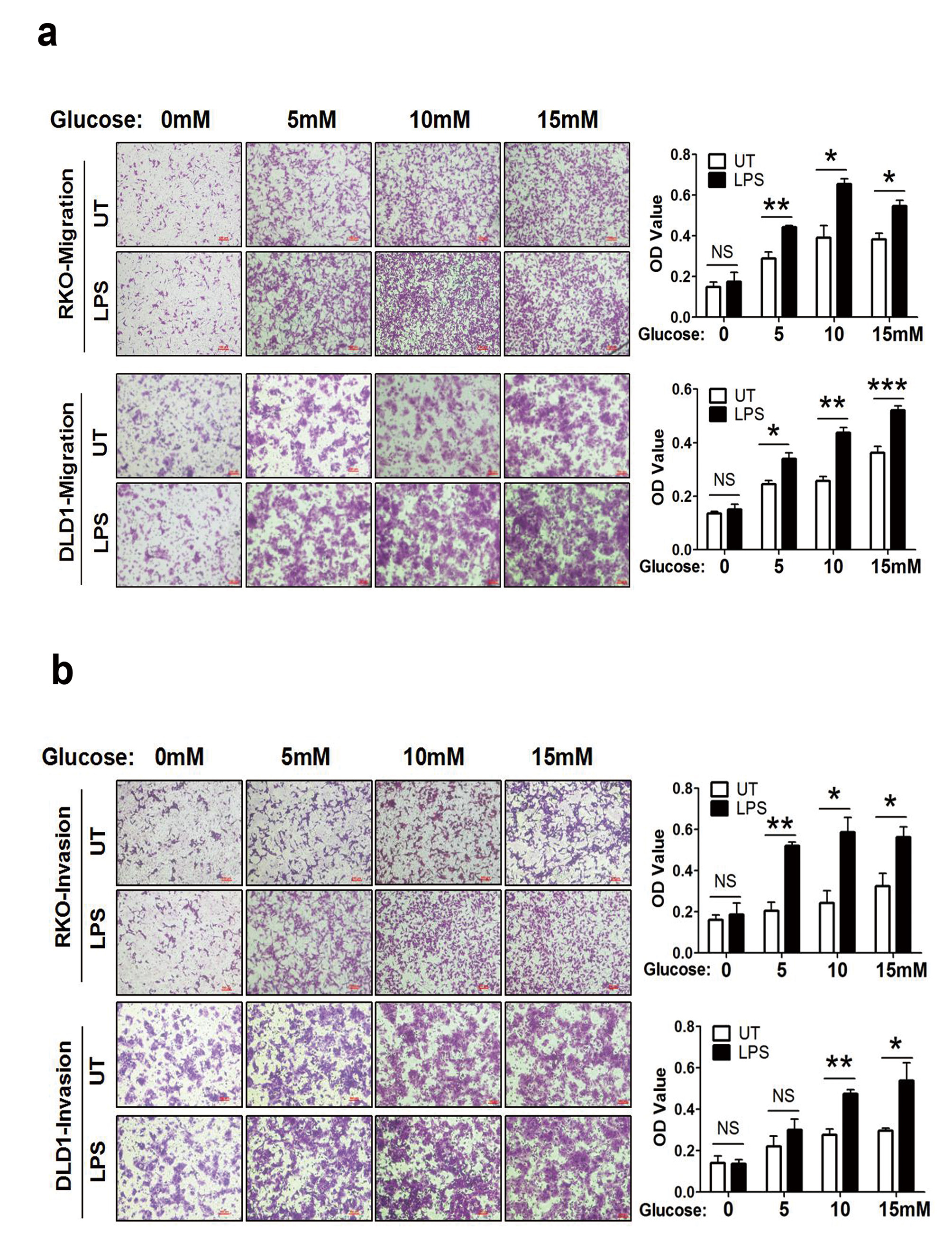

Supplement: Supplementary file 4 — Additional file 4: Supplementary Fig. 3. LPS promoted migration and invasion depending on glucose concentration. a. The effect of different glucose concentration on cell migration. Migration was determined by transwell assay. Cells were cultured with the indicated glucose concentrations (0, 5, 10, 15mM) for 24 hrs and stimulated with or without 1μg/ml LPS. b. The effect of different glucose concentration on cell invasion. Invasion was determined by transwell assay. Cells were cultured with the indicated glucose concentrations (0, 5, 10, 15mM) for 24 hrs and stimulated with or without 1μg/ml LPS. [file 40170_2021_260_MOESM4_ESM.jpg]
